# Synthesis & Evaluation of Novel Mannosylated Neoglycolipids for Liposomal Delivery System Applications

**DOI:** 10.3390/pharmaceutics14112300

**Published:** 2022-10-26

**Authors:** Leila Mousavifar, Jordan D. Lewicky, Alexis Taponard, Rahul Bagul, Madleen Rivat, Shuay Abdullayev, Alexandrine L. Martel, Nya L. Fraleigh, Arnaldo Nakamura, Frédéric J. Veyrier, Hoang-Thanh Le, René Roy

**Affiliations:** 1 Glycosciences and Nanomaterial Laboratory, Université du Québec à Montréal, P.O. Box 8888, Succ. Centre-Ville, Montréal, QC H3C 3P8, Canada; 2 Health Sciences North Research Institute, 56 Walford Road, Sudbury, ON P3E 2H2, Canada; 3 Armand-Frappier Santé Biotechnologie Research Centre, Institut National de la Recherche Scientifique, 531 Boulevard des Prairies, Laval, QC H7V 1B7, Canada; 4 Medicinal Sciences Division, NOSM University, 935 Ramsey Lake Road, Sudbury, ON P3E 2C6, Canada

**Keywords:** liposomes, mannose, drug delivery, dynantin

## Abstract

Glycosylated NPs, including liposomes, are known to target various receptors involved in cellular carbohydrate transport, of which the mannoside binding receptors are attracting particular attention for their expression on various immune cells, cancers, and cells involved in maintaining central nervous system (CNS) integrity. As part of our interest in NP drug delivery, mannosylated glycoliposomal delivery systems formed from the self-assembly of amphiphilic neoglycolipids were developed, with a C_12_-alkyl mannopyranoside (ML-C_12_) being identified as a lead compoundcapable of entrapping, protecting, and improving the delivery of structurally diverse payloads. However, ML-C_12_ was not without limitations in both the synthesis of the glycolipids, and the physicochemical properties of the resulting glycoliposomes. Herein, the chemical syntheses of a novel series of mannosylated neoglycolipids are reported with the goal of further improving on the previous ML-C_12_ glyconanoparticles. The current work aimed to use a self-contingent strategy which overcomes previous synthetic limitations to produce neoglycolipids that have one exposed mannose residue, an aromatic scaffold, and two lipid tails with varied alkyl chains. The azido-ending carbohydrates and the carboxylic acid-ending lipid tails were ligated using a new one-pot modified Staudinger chemistry that differed advantageously to previous syntheses. The formation of stable neoglycoliposomes of controllable and ideal sizes (≈100–400 nm) was confirmed via dynamic light scattering (DLS) experiments and transmission electron microscopy (TEM). Beyond chemical advantages, the present study further aimed to establish potential improvements in the biological activity of the neoglycoliposomes. Concanavalin A (Con A) agglutination studies demonstrated efficient and stable cross-linking abilities dependent on the length of the linkers and lipid tails. The efficacy of the glycoliposomes in improving cytosolic uptake was investigated using Nile Red as probe in immune and cancer cell lines. Preliminary ex vivo safety assessments showed that the mannosylated glycoliposomes are hemocompatible, and non-immunogenic. Finally, using a model peptide therapeutic, the relative entrapment capacity and plasma stability of the optimal glycoliposome delivery system was evaluated and compared to the previous neoglycoliposomes. Overall, the new lead glycoliposome showed improved biological activity over ML-C_12_, in addition to having several chemical benefits including the lack of stereocenters, a longer linker allowing better sugar availability, and ease of synthesis using novel one-pot modified Staudinger chemistry.

## 1. Introduction

Nanotechnology-based drug delivery systems have made striking progress owing to the exceptional physicochemical properties of nanoparticles (NPs) [1,2,3]. NPs and liposome drug delivery vehicles in particular have proven useful to increase the uptake of therapeutics into target cells, leading to higher intracellular drug concentrations, higher therapeutic efficacy, and lower off-target toxicity by altering their biodistribution. Liposomes are particularly interesting as NP delivery systems due to their amphiphilic nature that mimics the phospholipid membrane of cells that allows the incorporation of both hydrophilic and lipophilic drugs. Of the various nanoparticle-based drug delivery systems, liposomes and polymer NPs constitute the vast majority of Food and Drug Administration (FDA)- and European Medicine Agency-approved formulations [3].

Exploiting both the transcytosis capacity and variable degrees of expression of receptors expressed at the cell surface, the active targeting of therapeutic-loaded NPs for site- and cell-selective delivery can be achieved by attaching the respective ligands to the NP surface [4]. The list of receptors being investigated for active NP targeting continues to expand [5]. Sugars, including monosaccharides and disaccharides, have been used to form glycosylated NPs that target various receptors involved in cellular carbohydrate transport [6,7]. Mannose receptors are ubiquitous transmembrane glycoproteins that have been targeted to increase the affinity of therapeutic-loaded NPs to a variety of different cell types, including dendritic cells, macrophages and several different types of cancer [8,9,10,11,12,13,14,15]. The mannose receptor is also expressed on macrophages and microglia of the blood–brain barrier (BBB) [16], and mannosylated liposomes have been used to improve the uptake of a variety of different therapeutic agents into the central nervous system (CNS) [17,18,19,20,21].

**Figure 1:**
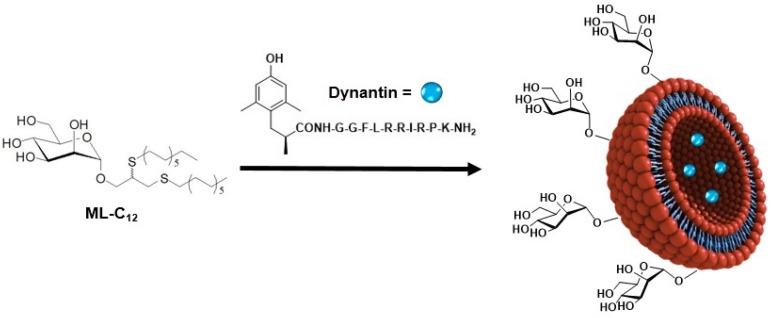
Schematic representation of mannosylated liposomes ML-C_12_ for the encapsulation of Dynantin.

As part of ongoing efforts towards the development of targeted NP delivery systems, a library of novel amphiphilic neoglycolipids that self-assemble to form monodisperse glycoliposomes when added to water have been previously reported [22]. Glycoliposomal NPs formed with a C_12_-alkyl-mannopyranoside member of the library (ML-C_12_, Figure 1) were able to entrap and protect different peptide-based therapeutics from degradation in plasma ex vivo [23]. It was shown that the ML-C_12_ glycoliposome system improved the delivery and activity of Dynantin (Figure 1), a synthetic dynorphin peptide analogue kappa opioid receptor (KOR) antagonist [24] into the CNS of mice in vivo when administered intranasally [25,26]. KOR antagonists, such as Dynantin, show promise in the treatment of addiction and its associated depression [27]. However, the use of Dynantin in a clinical setting has been prohibited due to pharmacokinetic limitations, including poor CNS penetration and plasma enzymatic degradation [28], that our mannosylated glycoliposomes are able to overcome [24,25].

In previous work, a straightforward chemical strategy to develop ML-C_12_ and other carbohydrate-based neoglycolipids was used [22]. Their preparation relied on the use of a sugar bearing alkyne functionality in the aglyconic portion. Sequential photo-catalyzed thiol-yne ligation led, when required, to either homo- or hetero-functionalized lipid tails [22]. One of the drawbacks of this chemistry was that it generated a new stereocenter that resulted in ML-C_12_ and related glycolipids being isolated as an inseparable mixture of diastereomers. Ultimately, this is not an ideal situation in terms of pharmacological development, where defined and exact stereochemistry is required. In addition, the synthetic strategy led to neoglycolipids having short linkers between the sugar head-groups and the lipid tails, which might impact the ultimate availability of the sugar residues for interaction with their cognate carbohydrate binding receptors. With respect to the physicochemical properties of liposomes assembled from ML-C_12_, even though they were useful toward the stabilization of entrapped peptides [23], the methodology necessitated cholesterol to further stabilize the liposomal delivery system [25].

With the goal of further improving the earlier ML-C_12_ glyconanoparticles, described herein are the chemical syntheses of a novel series of mannosylated neoglycolipids having one exposed sugar residue, an aromatic scaffold that is devoid of any stereocenters, and two lipid tails with varied alkyl chains that form liposomal nanocarriers of controllable sizes. We aimed to prepared these series of compounds using a self-contingent strategy to overcome the above noted limitations by incorporating an additional alkyl linker between the sugar moiety and the scaffold as it is generally well known that mannopyranoside receptors bind better when the sugar moiety possesses hydrophobic aglycones [29]. The aglycone lipid architecture consisted of a 3,5-dihydoxybenzoic acid framework possessing two alkyl chains of eight to sixteen carbons. The azido-ending carbohydrates and the carboxylic acid-ending lipid architecture were ligated using a novel one-pot modified Staudinger chemistry to generate glycolipid structures that were chemically stable for over a year when stored at 4 °C. The proposed chemical ligation offers several advantages over preceding work since it allows the introduction of a wide range of different hydrophilic carbohydrate moieties and other entities such as probes and charges. Importantly, it greatly favors the control of the anomeric configuration of the sugar residue in early precursors rather than in the final products.

Based on the improved chemistry described above, the novel glycoliposomes are hypothesized to have superior biochemical properties, which we further aimed to evaluate in the current study using a variety of assays. Dynamic light scattering (DLS) experiments confirmed the formation of stable neoglycoliposomes of approximately 100–400 nm. The physicochemical stability of the glycoliposomes were initially evaluated in biochemical settings using agglutination assays. Agglutination studies with the phytohemagglutinin Concanavalin A (ConA) demonstrated efficient and stable cross-linking abilities that were dependent on the length of the linkers and lipid tails. The efficacy of cytosolic entrapment of the delivery system was demonstrated in immune (JAWSII, J774A.1) and cancer (HCT 116, A2780) cell lines using Nile Red as probe. Preliminary ex vivo safety assessments showed that the mannosylated glycoliposomes were hemocompatible, and non-immunogenic. Finally, Dynantin was again used as a model therapeutic where the relative entrapment capacity and the plasma stability of the best-behaved delivery system were compared to previous neoglycoliposomes formed with ML-C_12_.

## 2. Materials and Methods

### 2.1. Chemistry

#### 2.1.1. General Methods

All reactions in organic medium were performed in standard oven dried glassware under an inert atmosphere of nitrogen using freshly distilled solvents stored over molecular sieves. Solvents were deoxygenated, when necessary, by bubbling nitrogen through the solution. All reagents were used as supplied without prior purification and obtained from Sigma-Aldrich Chemical Co. (Toronto, ON, Canada). Reactions were monitored by analytical thin-layer chromatography (TLC) using silica gel 60 F254 precoated plates (E. Merck, Darmstadt, Germany) and compounds were visualized by 254 nm light and/or by dipping into a mixture of sulfuric acid and methanol in water or into a mixture of KMnO_4_ and K_2_CO_3_ in water followed by gentle warming with a heat-gun. Purifications were performed by flash column chromatography using silica gel from Canadian Life Science (60 Å, 40–63 μm) (Peterborough, ON, Canada) with the indicated eluent. ^1^H-NMR and ^13^C-NMR spectra were recorded at 300 and/or 600 MHz, 75 and/or 150 MHz, respectively, on a Bruker spectrometer (300 MHz and 600 MHz) (Milton, ON, Canada) and Varian spectrometer (600 MHz) (Milton, ON, Canada). All NMR spectra were measured at 25 °C in indicated deuterated solvents and are shown in the accompanying Supplementary Materials. Proton and carbon chemical shifts (δ) are reported in ppm and coupling constants (J) are reported in Hertz (Hz). The resonance multiplicities in the ^1^H-NMR spectra are described as “s” (singlet), “d” (doublet), “t” (triplet), and “m” (multiplet) and broad resonances are indicated by “broad”. Residual protic solvent of CDCl_3_ (^1^H, 7.27 ppm; ^13^C, 77.0 ppm (central resonance of the triplet)), D_2_O (^1^H, 4.80 ppm and 30.9 ppm for CH_3_ of acetone for ^13^C spectra) were used as standard. Two-dimensional homonuclear correlation ^1^H-^1^H COSY and ^1^H-^13^C HSQC experiments were used to confirm NMR peak assignments. Letters are used for NMR assignment. High-resolution mass spectra (HRMS) were measured with an LC-MS-TOF (Liquid Chromatography Mass Spectrometry Time Of Flight) (Agilent Technologies) in positive electrospray mode by the analytical platform of UQAM. Either protonated molecular ions [M + nH]^n+^ or adducts [M + nX]^n+^ (X = Na, K, NH_4_) were used for empirical formula confirmation. Compound ML-C_12_ was prepared as previously described [22].

#### 2.1.2. Dynamic Light Scattering

The self-assembly of the neoglycoliposomes into vesicles and liposomes involved the injection method of their solution in a water miscible solvent such as ethanol or tetrahydrofuran (THF) into water or buffer [22]. This methodology has been shown to be efficient for the self-assembly of the previously described amphiphilic mannosylated neoglycolipids into monodisperse vesicles [30]. The resulting assemblies were first analyzed by dynamic light scattering (DLS) for size, polydispersity (PDI), and stability in time. For liposome size determination, solutions of the neoglycolipids (3.5 mg) in THF (1.1 mL) were then diluted in distilled water (2.2 mL) to provide final concentrations of 1.06 mg/mL, as these conditions were found optimal toward the targeted liposomal diameter.

Particle size distributions were measured in water using a Zetasizer^®^ Nano S90 from Malvern Instruments (UK) equipped with 4mW He-Ne laser 633 nm and avalanche photodiode positioned at 90° to the beam and temperature-controlled cuvette holder. Instrument parameters were determined automatically along with measurement times. Experiments were performed in triplicate at 25 °C.

Crosslinking studies were carried out in 1 mol/L phosphate-buffered saline (PBS) for the plant lectin Concanavalin A (Con A) (Sigma-Aldrich) known to bind multivalent mannosylated glycoconjugates [31] as previously described [22,32,33]. For this, ConA (0.5 mg) was dissolved in PBS buffer (0.5 mL); aliquots of 0.5 mL (~0.5 mg) were taken from the original lipososomal solutions described above and the size of the steadily increasing cross-linked aggregates were monitored as a function of time.

#### 2.1.3. Transmission Electron Microscopy

Stock solutions (25 µg/µL) of ML-C_12_ or **24** were prepared by dissolving the compounds (2.5 mg) in 100 µL of *tert*-Butanol (TBA, Alfa Aesar, Ottawa, ON, Canada) with heating at 100 °C for 5 min, and were stored at 4 °C. Aqueous solutions (0.5 mg/mL were prepared by adding the glycolipid stock solutions (2 µL) to ddH_2_O (100 µL), and were stores stored without filtration at room temperature for 24 h. On a parafilm, grids were immersed in a drop (50 µL) of the lipid nanoparticle formulations for 20 min. The grids were dried using a piece of bibulous paper, and then immersed in a drop of a 3% phosphotungstic acid solution (PTA, pH = 6.0) for 1 min. The grids were dried again, and the imaging was performed at 75 kV on a Hitachi-7100 transmission electron microscope (TEM, Hitachi Limited, Ibaraki, Japan) equipped with an XR-100 camera (Advanced Microscopy Techniques Corporation, Danvers, MA, USA).

#### 2.1.4. Chemical Syntheses

*Methyl 3,5-dihydroxybenzoate* (**2**). To a solution of 3,5-dihydroxybenzoic acid (**1**) (5.00 g, 32.4 mmol, 1 equiv.) in dry methanol (40 mL, 30.5 equiv.) was added conc. H_2_SO_4_ (0.25 mL, 0.15 equiv). The reaction mixture was heated at 70 °C for 7 h. The solution was evaporated under reduced pressure. The solid was treated carefully with a NaHCO_3_ solution and the product was extracted with EtOAc. The organic phase was washed with water and brine and finally evaporated to afford **2** (5.00 g, 29.7 mmol, 92%) as a white solid whose physical characteristics are in complete agreement with the published data [34]. ^1^H NMR (300MHz, (CD_3_)_2_SO)): δ 9.66 (s, 2H, OH), 6.85 (d, 2H, *J_ortho-para_* = 2.2 Hz, H_ortho_), 6.48 (t, 1H, *J_para-ortho_* = 2.2 Hz, H_para_), 3.83 (s, 3H, OCH_3_); ^13^C NMR (Bruker 75MHz, (DMSO-d_6_)): δ 166.4 (C_Ester_), 158.7 (C_Arom_), 131.5 (C_Arom_), 107.3 (x2, C_Arom_), 52.1 (C_OMe_).

##### General Procedure for methyl 3,5-bis(alkyloxy)benzoates (3-7)-Method A

The syntheses of this series of compounds followed our previously published protocols [30]. To a mixture of methyl 3,5-dihydroxybenzoate (**2**) (1.00 g, 5.95 mmol, 1 equiv.), K_2_CO_3_ (2.22 g, 16.1 mmol, 2.7 equiv.) and KI (69 mg, 417 nmol, 0.07 equiv.) in DMF (25 mL) was added the corresponding 1-bromoalkanes (2.76 g, 14.28 mmol, 2.4 equiv.). The reaction mixture was stirred at 90 °C for 24 h. It was cooled and diluted with DCM (100 mL). The solution was washed with water (2 × 15 mL) and brine (15 mL) and the organic layer was dried over anhydrous Na_2_SO_4_. The solvent was removed and a solid residue was obtained. The solid was filtered and washed with methanol and dried. The crude products were further purified by passing through a column of silica gel (hexanes, 1–4% EtOAc in hexanes as eluents) yielding (**3–7**) as white solids.

*Methyl 3,5-bis(octyloxy)benzoate* (**3**). White solid (1.19 g, 30 mmol, 51%). ^1^H NMR (300MHz, CDCl_3_): δ 7.16 (d, J = 3 Hz, 2H, H1), 6.63 (t, J = 3 Hz, 1H, H2), 3.96 (t, J = 6 Hz, 4H, H3), 3.89 (s, 3H, OCH_3_), 1.77 (tt, J = 9 Hz, J = 6 Hz, 4H, H4), 1.45–1.29 (m, 20H, H5), 0.89 (t, J = 6 Hz, 6H, H6). ^13^C NMR (Bruker 300MHz, CDCl_3_): δ 167.1 (C_Ester_), 160.3 (C_Ar_), 131.9 (C_Ar_), 107.7 (C_Ar_), 106.7 (C_Ar_), 68.4, 52.3, 31.9, 29.5, 29.4, 29.3, 26.2, 22.8, 14.3 (CH_3_).

*Methyl 3,5-bis(decyloxy)benzoate* (**4**). White solid (2.00 g, 4.60 mmol, 75%). ^1^H NMR (300MHz, CDCl_3_): δ 7.16 (d, *J* = 3 Hz, 2H, H1), 6.63 (t, *J* = 3 Hz, 1H, H2), 3.97 (t, *J* = 6 Hz, 4H, H3), 3.90 (s, 3H, OCH_3_), 1.77 (tt, *J* = 9 Hz, *J* = 6 Hz, 4H, H4), 1.45–1.27 (m, 28H, H5), 0.88 (t, *J* = 6 Hz, 6H, H6). ^13^C NMR (300MHz, CDCl_3_): δ 167.1 (C_Ester_), 160.3 (C_Ar_), 131.9 (C_Ar_), 107.7 (C_Ar_), 106.7 (C_Ar_), 68.4, 52.3, 32.0, 29.7 (x2), 29.5 (x2), 29.3, 26.1, 22.8, 14.2 (CH_3_).

*Methyl 3,5-bis(dodecyloxy)benzoate* (**5**). White solid (2.37 g. 4.70 mmol, 78%). ^1^H-NMR (300MHz, CDCl_3_): δ 7.15 (d, *J* = 3 Hz, 2H, H1), 6.63 (t, *J* = 3 Hz, 1H, H2), 3.96 (t, *J* = 6 Hz, 4H, H3), 3.89 (s, 3H, OCH_3_), 1.77 (tt, *J* = 9 Hz, *J* = 6 Hz, 4H, H4), 1.45–1.27 (m, 36H, H5), 0.88 (t, *J* = 6 Hz, 6H, H6); ^13^C NMR (300MHz. CDCl_3_): δ 167.1 (C_Ester_), 160.3 (C_Ar_), 132.0 (C_Ar_), 107.8 (C_Ar_), 106.7 (C_Ar_), 68.5, 52.3, 32.1, 29.8 (x2), 29.7 (x2), 29.5, 29.3, 26.2, 22.8, 14.3 (CH_3_).

*Methyl 3,5-bis(tetradecyloxy)benzoate* (**6**). White solid (4.97 g. 9.09 mmol, 91%). ^1^H NMR (300MHz. CDCl_3_): δ 7.16 (d, *J* = 3 Hz, 2H, H1), 6.63 (t, *J* = 3 Hz, 1H, H2), 3.96 (t, *J* = 6 Hz, 4H, H3), 3.89 (s, 3H, OCH_3_), 1.77 (tt, *J* = 9 Hz, *J* = 6 Hz, 4H, H4), 1.44–1.26 (m, 44H, H5), 0.88 (t, *J* = 6 Hz, 6H, H6); ^13^C NMR (300MHz. CDCl_3_): δ 167.1 (C_Ester_), 160.3 (C_Ar_), 131.9 (C_Ar_), 107.7 (C_Ar_), 106.7 (C_Ar_), 68.4, 52.3, 32.1, 29.8 (x3), 29.7, 29;5, 29.3, 26.2, 22.8, 14.3 (CH_3_).

*Methyl 3,5-bis(hexadecyloxy)benzoate* (**7**). White solid (2.68 g. 4.48 mmol, 73%). ^1^H NMR (300 MHz, CDCl_3_): δ 7.15 (d, *J* = 3 Hz, 2H, H1), 6.63 (t, *J* = 3 Hz, 1H, H2), 3.96 (t, *J* = 6 Hz, 4H, H3), 3.90 (s, 3H, OCH_3_), 1.77 (tt, *J* = 9 Hz, *J* = 6 Hz, 4H, H4), 1.45–1.26 (m, 52H, H5), 0.88 (t, *J* = 6 Hz, 6H, H6); ^13^C NMR (Bruker 300 MHz, CDCl_3_) δ 167.1 (C_Ester_), 160.3 (C_Ar_), 131.9 (C_Ar_), 107.8 (C_Ar_), 106.7 (C_Ar_), 68.4, 52.3, 32.1, 29.9, 29.8 (x2), 29.7, 29.5, 29.3, 26.2, 22.8, 14.3 (CH_3_).

##### General Procedure for Hydrolysis of Methyl Ester (8–12)-Method B

The syntheses of this series of compounds followed our previously published protocols [30]. A solution of methyl 3,5-bis(alkyloxy)benzoate (**3**–**7**) (1.00 g, 2.55 mmol, 1 equiv.) with KOH (595 mg, 10.6 mmol, 4.2 equiv.) in water (7 mL) and ethanol (40 mL) were refluxed for 4 h. Then, reaction mixture was cooled to room temperature and concentrated hydrochloric acid was added carefully until pH = 1. CH_2_Cl_2_ and water were added and the combined organic fractions were separated, dried over MgSO_4_ and evaporated to yield 3,5-bis(alkyloxy)benzoic acid **8**–**12**.

*3,5-Bis(octyloxy)benzoic acid* (**8**). White solid (855 mg. 87%). ^1^H NMR (300 MHz, CDCl_3_): δ 7.23 (d, *J* = 3 Hz, 2H, H1), 6.69 (t, *J* = 3 Hz, 1H, H2), 3.98 (t, *J* = 6 Hz, 4H, H3), 1.79 (tt, *J* = 9 Hz, *J* = 6 Hz, 4H, H4), 1.46–1.25 (m, 20H, H5), 0.89 (t, *J* = 6 Hz, 6H, H6); ^13^C NMR (300 MHz, CDCl_3_): δ 172.5 (C_CO2H_), 160.4 (C_Ar_), 131.1 (C_Ar_), 108.3 (C_Ar_), 107.6 (C_Ar_), 68.5, 32.0, 29.5, 29.4, 29.3, 26.2, 22.9, 14.2 (CH_3_).

*3,5-Bis(decyloxy)benzoic acid* (**9**). White solid (1.31 g, 90%). ^1^H NMR (300 MHz, CDCl_3_): δ 7.22 (d, *J* = 3 Hz, 2H, H1), 6.69 (t, *J* = 3 Hz, 1H, H2), 3.98 (t, *J* = 6 Hz, 4H, H3), 1.79 (tt, *J* = 9 Hz, *J* = 6 Hz, 4H, H4), 1.48–1.27 (m, 28H, H5), 0.89 (t, *J* = 6 Hz, 6H, H6); ^13^C NMR (300 MHz, CDCl_3_): δ 172.4 (C_CO2H_), 160.4 (C_Ar_), 131.1 (C_Ar_), 108.3 (C_Ar_), 107.6 (C_Ar_), 68.5, 32.1, 29.7 (x2), 29.5 (x2), 29.3, 26.2, 22.8, 14.3 (CH_3_).

*3,5-Bis(dodecyloxy)benzoic acid* (**10**). White solid (2.08 gm, 97%). ^1^H NMR (300 MHz, CDCl_3_) δ 7.22: (d, *J* = 3 Hz, 2H, H1), 6.69 (t, *J* = 3 Hz, 1H, H2), 3.98 (t, *J* = 6 Hz, 4H, H3), 1.79 (tt, *J* = 9 Hz, *J* = 6 Hz, 4H, H4), 1.46–1.27 (m, 36H, H5), 0.88 (t, *J* = 6 Hz, 6H, H6); ^13^C NMR (300 MHz, CDCl_3_): δ 172.4 (C_CO2H_), 160.4 (C_Ar_), 131.1 (C_Ar_), 108.3 (C_Ar_), 107.6 (C_Ar_), 68.5, 32.1, 29.8 (x3), 29.7, 29.5, 29.3, 26.2, 22.9, 14.3 (CH_3_).

*3,5-Bis(tetradecyloxy)benzoic acid* (**11**). White solid (1.03 g, 69%). ^1^H NMR (300 MHz, CDCl_3_): δ 7.22 (d, *J* = 3 Hz, 2H, H1), 6.69 (t, *J* = 3 Hz, 1H, H2), 3.98 (t, *J* = 6 Hz, 4H, H3), 1.79 (tt, *J* = 9 Hz, *J* = 6 Hz, 4H, H4), 1.46–1.26 (m, 44H, H5), 0.88 (t, *J* = 6 Hz, 6H, H6); ^13^C NMR (300 MHz, CDCl_3_): δ 172.4 (C_CO2H_), 160.4 (C_Ar_), 131.1 (C_Ar_), 108.3 (C_Ar_), 107.6 (C_Ar_), 68.5, 32.1, 29.8 (x2), 29.7, 29.5, 29.3, 26.2, 22.9, 14.3 (CH_3_).

*3,5-Bis(hexadecyloxy)benzoic acid* (**12**). White solid (1.43 g, 97%). ^1^H NMR (300 MHz, CDCl_3_): δ 7.22 (d, J = 3 Hz, 2H, H1), 6.68 (t, *J* = 3 Hz, 1H, H2), 3.98 (t, *J* = 6 Hz, 4H, H3), 1.78 (tt, *J* = 9 Hz, *J* = 6 Hz, 4H, H4), 1.45–1.26 (m, 52H, H5), 0.88 (t, *J* = 6 Hz, 6H, H6); ^13^C NMR (300 MHz, CDCl_3_) δ 172.4 (C_CO2H_), 160.4 (C_Ar_), 131.1 (C_Ar_), 108.3 (C_Ar_), 107.6 (C_Ar_), 68.5, 32.1, 29.9 (x2), 29.8 (x2), 29.5, 29.3, 26.2, 22.9, 14.3 (CH_3_).

##### General Procedure for the Carbohydrate Synthesis-Method C

*6-Chlorohexyl tetra-O-acetyl-α-D-mannopyranoside* (**14**). Mannose pentaacetate (**13**) (507 mg. 1.30 mmol. 1.0 eq.) and 6-chlorohexanol (340 µL, 2.56 mmol. 2.0 eq.) were dissolved in dry DCM (10 mL) at 0 °C. Et_2_O.BF_3_ (480 µL, 3.90 mmol. 3.0 eq.) was added dropwise at 0 °C then at 40 °C for 17 h. The solution was neutralized by adding a saturated solution of NaHCO_3_ (10 mL). The organic phase was extracted with dichloromethane, washed with water and brine. The crude product was dried over sodium sulfate, filteredand the solvent was removed under low pressure. The product was purified by column chromatography (Gradient Hexane: EtOAc. 100:0 to 70:30) to afford pure 6-chlorohexyl tetra-O-acetyl-α-D-mannopyranoside (**14**) as a yellow oil (404 mg. 66%). ^1^H NMR (300 MHz, CDCl_3_): δ 5.34 (dd, *J* = 9 Hz, *J* = 3 Hz, 1H), 5.30–5.21 (m, 2H), 4.80 (d, *J* = 3 Hz, 1H), 4.28 (dd, *J* = 12 Hz, *J* = 3 Hz, 2H), 4.12–4.08 (m, 1H), 4.00–3.94 (m, 1H), 2.15 (s, 3H, OAc), 2.10 (s, 3H, OAc), 2.04 (s, 3H, OAc), 1.99 (s, 3H, OAc), 1.81–1.74 (m, 2H), 1.62–1.48 (m, 2H), 1.50–1.37 (m, 4H); ^13^C NMR (300 MHz, CDCl_3_): δ 170.6–169.9 (4 x CO_Ac_), 97.2, 69.7, 69.7, 68.4 (CH_2_-O), 68.1, 66.1, 62.5, 45.0, 32.5 (CH_2_), 29.1 (CH_2_), 26.2 (CH_2_), 25.4 (CH_2_), 20.9–20.7 (4 x OAc). Data agreed with those of the literature [35,36,37].

*6-Azidohexyl tetra-O-acetyl-α-D-mannopyranoside* (**15**). The 6-chlorohexyl 2,3,4,6-tetra-*O*-acetyl-α-D-mannopyranoside (**14**) (551 mg, 1.18 mmol, 1.0 equiv.) was dissolved in dry DMF (5 mL). Sodium azide (385 mg, 5.92 mmol, 5.0 equiv.) and sodium iodide (40 mg, 0.22 equiv.) were added to the mixture. The solution was stirred at 80 °C for 24 h. The mixture was diluted with water and the aqueous phase was extracted 3 times with EtOAc. The organic phase was washed with water, brine, then dried over sodium sulfate, filtered and concentrated under reduced pressure. The product was purified by silica gel column chromatography (Toluene, EtOAc, 3:2) to yield pure title compound **(15**) as a yellow oil (521 mg, 1.10 mmol, 93%); *R_f_* = 0.67 (Toluene, EtOAc, 3:2). IR: (2104 cm^−1^ for N_3_). ^1^H NMR (300 MHz, CDCl_3_): δ 5.36 (dd, 1H, *J*_2,3_ = 3.3 Hz, *J*_3,4_ = 10 Hz, H-3), 5.32–5.22 (m, 2H, H-4, H-2), 4.81(d, 1H, *J*_1,2_ = 1.6 Hz, H-1), 4.29 (dd, 1H, *J*_6a, 6b =_ 12.2 Hz, *J*_5, 6a *=*_ 5.3 Hz, H-6a), 4.09 (dd, 1H, *J*_6a, 6b_ = 12.2 Hz, *J*_5, 6b_ = 2.4 Hz, H-6b), 3.98 (ddd, 1H, *J*_4, 5_ = 9.4 Hz, *J*_5, 6a_ = 5.3 Hz, *J*_5, 6b_ = 2.4 Hz, H-5), 3.76–3.66 (m, 1H, OC*H*H), 3.49- 3.42 (m, 1H, OCH*H*), 3.29 (t, 1H, *J* = 6.8 Hz, -CH_2_-N_3_), 2.15–1,99 (s, 12H, OAc), 1.73–1.50 (m, 4H), 1.50–1.29 (m, 4H); ^13^C NMR (CDCl_3,_): 170.6–169.7 (4×CO), 97.6 (CH_1_), 69.7, 69,1, 68.4, 68.3, 66.3, 62.5, 51.3 (CH_2_-N_3_), 29.1 (CH_2_), 28.7 (CH_2_), 26.5 (CH_2_), 25.7 (CH_2_), 20. 9–20.7 (4xOAc).

##### General Procedure for Synthesis of Mannolipids (16–20)-Method D

To a solution of 6-azidohexyl tetra-O-acetyl-α-D-mannopyranoside **15** (99 mg, 0.210 mmol, 1.0 equiv.) and derivatives of bis (alkyloxy) benzoic acid, (**8**–**12**) (81 mg, 0.213 mmol, 1.0 equiv.) in DCM (8.0 mL) was added tributylphopshine (70 µL, 0.312 mmol, 1.5 equiv.) at 0 °C. The mixture was stirred at 0 °C for 2 h and then at the room temperature for 24 h. The presence of amine was confirmed by TLC. To reaction mixture hydroxybenzotriazole (HOBT) (44 mg, 0.326 mmol,1.6 equiv.) and N, N’-diisopropylcarbodiimide (DIC) (70 µL, 0.45 mmol, 2.1 equiv.) were added and was stirred at room temperature for 96 h. The reaction was then diluted in ethyl acetate and the organic phase was washed with saturated NaHCO_3_ solution and brine, dried and concentrated. The crude product was purified by column chromatography (Hexane: EtOAc 1:0 to 7:3) to obtain compounds (**16**–**20**).

*N-[O-(2.3.4.6-Tetra-O-acetyl-α-D-mannopyranosyl)oxyhexyl]-3.5-bis(octyloxy)benzamide* (**16**). The compound was prepared according to general procedure **D** as a colorless oil compound (127 mg, 0.157 mmol. 74%). ^1^H NMR (300 MHz, CDCl_3_): δ 6.87 (d. 2H. *J_ortho-para_* = 2.2 Hz, H_ortho_), 6.55 (t, 1H, *J_para-ortho_* = 2.2 Hz, H*para*), 6.22 (t, 1H, *J* = 5.6 Hz, NH), 5.35 (dd, 1H, *J_H3-H4_* = 10 Hz, *J*_H3-H2_ = 3.3Hz, H_3_), 5.29 (d, 1H. *J_H4-H3_* = 9.6 Hz, H_4_), 5.23 (dd, 1H, *J_H2-H3_* = 3.2 Hz, *J*_H2-H1_ = 1.7 Hz, H_2_), 4.81 (d, 1H, *J_H1-H2_* = 1.3 Hz, H_1_), 4.29 (dd, 2H, *J_H6b-H6a_* = 12.2 Hz, *J_H6a-H5_* = 5.3 Hz, H6a), 4.11 (dd, 1H, *J_H6b-H6a_* = 12.2 Hz. *J_H6b-H5_* = 2.3 Hz, H_6b_), 4.01–4.00 (m, 1H, H5), 3.96 (t, 4H, *J_Hg-Hh_* = 6.5 Hz, H_g_), 3.73–3.66 (m, 1H, H_a_), 3.49–3.40 (m, 3H, H_a,_ H_f_), 2.16–2.00 (4s, 12H, OAc), 1.81–1.72 (m, 4H, H_h_), 1.68–1.61 (m, 4H, H_b,_ H_e_), 1.44–1.42 (m, 8H, H_c_, H_d_, H_i_), 1.31–1.28 (m, 16H, H_j_-H_m_), 0.89 (t, 6H, *J_Hn-Hm_* = 6.7 Hz, H_n_) (Figure S1).

*N-[O-(2.3.4.6-tetra-O-acetyl-α-D-mannopyranosyl)oxyhexyl]-3.5-bis(decyloxy)benzamide* (**17**). The compound was prepared according to general procedure **D** (155 mg. 0.168 mmol. 47%).^1^H-NMR (Bruker 300MHz, CDCl_3_): δ 6.86 (d. 2H. *^4^J_Hortho-Hpara_* = 2.1Hz. H_ortho_). 6.52 (t. 1H. *^4^J_Hpara-Hortho_* = 2.1Hz. H_para_). 6.34 (t. 1H. *J_Hamide-Hf_* = 5.7Hz. H_amide_). 5.33 (dd. 1H. *J_H3-H4_* = 10Hz. *J*_H3-H2_ = 3.3Hz. H_3_). 5.27 (d. 1H. *J_H4-H3_* = 9.6Hz. H_4_). 5.21 (dd. 1H. *J_H2-H3_* = 3.2Hz. J_H2-H1_ = 1.7Hz. H_2_). 4.78 (d. 1H. *J_H1-H2_* = 1.3Hz. H_1_). 4.27 (dd. 2H. *^2^J_H6b-H6a_* = 12.2Hz. *J’_H6a-H5_* = 5.3Hz. H_6a_). 4.08 (dd. 1H. *^2^J_H6b-H6a_* = 12.2Hz. *J’_H6b-H5_* = 2.3Hz. H_6b_). 3.99–3.97 (m. 1H. H_5_). 3.93 (t. 4H. *J_Hg-Hh_* = 6.5Hz. H_g_). 3.71–3.63 (m. 1H. H_a_). 3.47–3.37 (m. 3H. H_a_’. H_f_). 2.13–1.97 (s. 12H, 4x OAc). 1.76–1.69 (m. 4H. H_h_). 1.60–1.58 (m. 4H. H_b_. H_e_). 1.41–1.39 (m. 8H. H_c_. H_d_. H_i_). 1.27–1.24 (m. 32H. H_j_-H_q_). 0.86 (t. 6H. *J_Hr-Hq_* = 6.6Hz. H_r_) (Figure S2). ^13^C-NMR (Bruker 75MHz, CDCl_3_) δ 167.7 (Ca_mide_), 160.4 (C_Arom_), 136.6 (C_Arom_), 105.4 (C_Arom_), 104.4 (C_Arom_), 68.3, 32.0, 29.79, 29.75, 29.71, 29.5, 29.4, 29.3, 26.1, 22.7, 14.2 (CH3) (Figure S3). LC-MS-TOF: m/z found for [M+H]^+^ 752.5661, m/z calcd for C_43_H_77_NO_9_ [M+H]^+^ 752.5632.

*N-[O-(2.3.4.6-tetra-O-acetyl-α-D-mannopyranosyl)oxyhexyl]-3.5-bis(dodecyloxy)benzamide* (**18**). The compound was prepared according to general procedure **D** as a white solid (221 mg, 0.255 mmol, 78%). ^1^H NMR (Bruker 300 MHz, CDCl_3_) δ 6.89 (d, *^4^J_Hortho-Hpara_* = 2.3 Hz, H_ortho_), 6.56 (t, *^4^J_Hortho-Hpara_* = 2.2 Hz, H_para_), 5.32 (dd, J_H3-H4_ = 9.9, J_H3-H2_ = 3.3 Hz, H3), 5.29–5.16 (m, H4, H2), 4.78 (d, *J*_H1-H2_ = 1.8 Hz, H1), 4.26 (dd, *^2^J_H6b-H6a_* = 12.2 Hz, J_H6a-H5_ = 5.3 Hz, H6), 4.09 (dd, *J*_H6b-H6a_ = 12.2 Hz, J_H6b-H5_ = 2.5 Hz, H6b), 3.98–3.91 (m, H5, Hg), 3.67 (dt, *J* = 9.7, 6.5 Hz, Ha), 3.43 (dt, *J* = 9.4 Hz, 6.4 Hz, Ha’), 3.26 (t, J = 6.8 Hz, Hf), 2.13, 2.08, 2.02, 1.97 (s, 12H, 4x OAc), 1.74 (p, *J* = 6.8 Hz, Hh), 1.68–1.52 (m, Hb, Hf), 1.47–1.19 (m, Hc, Hd, Hi, Hj, Hk, Hl, Hm, Hn, Ho), 0.86 (t, *J* = 6.9 Hz, Hp) (Figure S4).

*N-[O-(2.3.4.6-tetra-O-acetyl-α-D-mannopyranosyl)oxyhexyl]-3.5-bis(tetradecyloxy)benzamide*(**19**). The compound was prepared according to general procedure **D** as a white solid (132 mg, 0.138 mmol. 61%). ^1^H NMR (Bruker 300 MHz, CDCl_3_): δ 6.87 (d, 2H, J_ortho-para_ = 2.2 Hz, H_ortho_), 6.55 (t, 1H, J_para-ortho_ = 2.2 Hz, H_para_), 6.20 (t, 1H, J = 5.7 Hz, NH), 5.36 (dd, 1H, J_3,4_ = 10 Hz, J_2,3_ = 3.3 Hz, H_3_), 5.30 (d, 1H, J_3,4_ = 9.7 Hz, H_4_), 5.21 (dd, 1H, J_2–3_ = 3.2 Hz, J_1,2_ = 1.7 Hz, H_2_), 4.81 (d, 1H, J_1–2_ = 1.7 Hz, H_1_), 4.29 (dd, H, J_6a,6b_ = 12. 2 Hz, J_5,6a_ = 5.3 Hz, H_6a_), 4.11 (dd, 1H, J_6a-6b_ = 12.2 Hz, J_5,6b_ = 2.4 Hz. H_6b_), 4.00–4.01 (m, 1H, H_5_), 3.97 (t, 4H, *J_g,h_* = 6.5 Hz. H_g_), 3.74–3.66 (m, 1H, H_a_), 3.50–3.40 (m, 3H, H_a’_, H_f_), 2.16–2.00 (s, 12H, 4x OAc), 1.82–1.72 (m, 4H, H_h_), 1.63–1.61 (m, 4H, H_b,_ H_e_), 1.44–1.42 (m, 8H, H_c,_ H_d,_ H_i_), 1.31–1.27 (m, 40H, H_j,_H_s_), 0.88 (t, 6H. J_t,-s_ = 6.7 Hz, H_t_); ^13^C NMR (75 MHz, CDCl_3_): δ 170.5–169.7 (CO_OAc_), 160.3 (C_amide_), 136.8 (C_Arom_), 105.2 (C_Arom_), 104.0 (C_Arom_), 69.7, 69.1, 68.4, 68.2, 66.2, 62.5, 31.9, 29.7, 29.6, 29.6, 29.5, 29.37, 29.35, 29.2, 26.0, 22.6, 20.8–20.6 (4xs, O-CO-CH_3_), 14.1 (Figure S5).

*N-[O-(2.3.4.6-tetra-O-acetyl-α-D-mannopyranosyl)oxyhexyl]-3.5-bis(hexadecyloxy)benzamide* (**20**). Prepared according to general procedure **D** as a white solid (22.6 mg, 0.022 mmol, 66%).^1^H NMR (300 MHz, CDCl_3_) δ 6.88 (d, J_ortho,para_ = 2.0 Hz, 2H_ortho_), 6.56 (d, J_para,ortho_ = 2.2 Hz, 1H_para_), 6.17 (s, NH), 5.36 (dd, 1H, J_3,4_ = 10.0 Hz, J_2,3_ = 3.3 Hz, H_3_), 5.30 (dd, 1H, J_3,4_ = 9.6Hz, H_4_), 5.24 (dd, 1H, J_2,3_ = 3.2Hz, J_1,2_ = 1.7 Hz, H_2_), 4.81 (d, 1H, J_1, 2_ = 1.5 Hz, H_1_), 4.30 (dd,1H, J_6a,6b_ = 12.3 Hz, J_5,6a_ = 5.4 Hz, H_6a_), 4.12 (dd, 1H, J_6a,6b_ = 12.3 Hz, J_5,6b_ = 2.4 Hz, H_6b_), 3.99–3.95 (m, 5H, H_5_, H_g_), 3.78–3.66 (m, 1H, H_a_), 3.46 (m, 3H, H_a’_, H_f_-H_f’_). 2.17,2.11, 1.05, 2,01 (s, 12H, 4 x OAc), 1.82–1.73 (m, 4H, H_h_), 1.68–1.61(m, 8H, H_z_, H_b_, H_e_), 1.5–1.4 (m, 8H, H_c_, H_d_, H_i_), 1.4–1.2 (m, 44H, H_j_-H_t_), 0.91–0.87 (t, 6H, *J_u,v_* = 6.7Hz, H_u_, H_v_, H_w_) (Figures S6 and S7).

##### General Procedure for de-O-Acetylation of Compounds (21–25)-Method E

To a solution of (1 eq.) of compounds (**16**–**20**) in dry MeOH (2 mL) was added NaOMe solution in 25 wt.% in MeOH (0.5 eq.) at room temperature under a nitrogen atmosphere. After 3h of stirring, the reaction mixture was neutralized with acidic resin Amberlite^®^ IR120 H^+^, filtered over a pad of celite and concentrated in vacuo then the solvent was removed in vacuo. The residue was then lyophilized to yield the fully deprotected final products (**21**–**25**).

The compounds were stored at −20 °C for at least 1 year before performing the bioactivity tests.

*N-[O-(α-D-mannopyranosyl)oxyhexyl]-3.5-bis(octyloxy)benzamide* (**21**). The compound was prepared according to general procedure **E** as a white solid (92 mg, 0,144 mmol, 92%). ^1^H-NMR (300 MHz, CDCl_3_) δ 6.88 (d, 2H, *J_Hortho-Hpara_* = 2.0 Hz, H_ortho_), 6.85 (t, 1H, *J* = 5.8, NH), 6.51 (t, 1H, *J_ortho-para_* = 2.3 Hz, H_para_), 4.79 (s, 1H, H_1_), 3.90–3.35 (14H, H_3_, H_4_, H_2_, H_6a_, H_6b_, H_5_, H_g_, H_a_, H_a’_, H_f_), 1.73–1.66 (m, 4H, H_h_), 1.54 (4H, H_b_, H_e_), 1.38–1.25 (24H, H_c_, H_d_, H_i_-H_m_), 0,87 (t, 6H, *J_Hn-Hm_* = 6_._6Hz, H_n_); ^13^C-NMR (75 MHz, CDCl_3_) δ 167.7 (C_amide_), 160.3 (C_meta_), 136.6 (C_ipso_), 105.4 (C_ortho_), 104.4 (C_para_), 100.1 (C_1_)_,_ 68.3, 31.9, 29.7, 29.6, 29.4, 29.3, 29.2, 26.1, 22,7, 14.1 (CH_3_) (Figure S8). LC-MS-TOF: *m/z* found [M+H]^+^ 640.4423, *m/z* calcd for C_35_H_61_NO_9_ [M+H]^+^ 640.4380.

*N-[O-(α-D-mannopyranosyl)oxyhexyl]-3.5-bis(decyloxy)benzamide* (**22**). The compound was prepared according to general procedure **E** (141 mg, 0.202 mmol, 98%) as a white solid. ^1^H NMR (300 MHz, CDCl_3_) δ 6.86 (d, 2H, J_ortho,para_ = 2.3 Hz, H_or__tho_), 6.64 (t, J = 5.8 Hz, NH), 6.51 (t, J_ortho,para_ *_=_* 2.2 Hz, H_para_), 4.83–4.73 (m, 1H, H_1_, 4 x OH), 3.95–3.46 (m, H_3_, H_4,_ H_g_, H_2_, H_5_, H_6,_ H_6′_, H_a_), 3.39–3.33 (m, H_a’_, H_f_), 1.71 (p, J = 6.7 Hz, H_h_), 1.69–1.42 (m, H_i_, H_e_), 1.47–1.07 (m, H_b_, H_c_, H_d_, H_j_, H_k_, H_l_, H_m_, H_n_, H_o_), 0.86 (t, 1H, H_p_) (Figure S9); ^13^C NMR (75 MHz, CDCl_3_) δ 167.7 (C_amide_), 160.4 (C_meta_), 136.6 (C_ipso_), 105.4 (C_ortho_), 104.3 (C_para_), 100.1 (C_1_), 72.4, 71.7, 71.1, 68.3 (C_g_), 67.7, 66.4, 61.1, 51.4, 40.2, 32, 29.6, 29.5, 29.5, 29.4, 29.3, 29.3, 28.8, 26.8, 26.6, 26.1, 25.9, 25.8, 22.7, 14.2 (Figure S10). LS-MS-TOF: *m/z* found [M+H]^+^ 696.5042, *m/z* calcd for C_39_H_69_NO_9_ [M+H]^+^ 696.5045.

*N-[O-(α-D-mannopyranosyl)oxyhexyl]-3.5-bis(dodecyloxy)benzamide* (**23**). The compound was prepared according to general procedure **E** as a white solid (89 mg, 0.118 mmol, 70%). ^1^H NMR (300 MHz, CDCl_3_) δ 6.88 (d, 2H, J_ortho,para_ = 2.3 Hz, H_o_), 6.77 (1H, J = 5.8 Hz, NH), 6.51 (t, J_ortho,para_ *_=_* 2.1 Hz, H_para_), 4.80 (s, 1H, H_1_), 3.90–3.36 (14H, H_3_, H_4_, _H2_, H_6a_, H_6b_, H_5_, H_g_, H_a_, H_a’_, H_f_), 1.74–1.69 (m, 4H, H_h_), 1.55 (4H, H_b_, H_e_), 1.32–1.26 (40H, H_c_, H_d_, H_i_-H_q_), 0.88 (t, 6H, J_r,q_ = 6.4Hz, Hr); ^13^C NMR (75 MHz, CDCl_3_) δ 167.7 (C_amide_), 160,4 (C_meta_), 136.6 (C_ipso_), 105.4 (C_ortho_), 104.4 (C_para_), 100.1 (C_1_), 68.3, 32.0, 29.79, 29.75, 29.71, 29.5, 29.4, 29.3, 26.1, 22.7, 14.2 (Figure S11). LC-MS-TOF: *m/z* found [M+H]^+^ 752.5661, *m/z* calcd for C_43_H_77_NO_9_ [M+H]^+^ 752.5632.

*N-[O-(α-D-mannopyranosyl)oxyhexyl]-3.5-bis(tetradecyloxy)benzamide* (**24**). The compound was prepared according to general procedure **E** as a white solid (84 mg, 0.104 mmol, 77%). ^1^H NMR (300 MHz, CDCl_3_) δ 6.87 (d, 2H, J_ortho,para_ = 1.8Hz, H_ortho_), 6.70 (t, 1H, J = 5.3Hz, NH), 6.51 (t, J_ortho,para_ *_=_* 2.1 Hz, H_para_), 5.11–4.98 (m, 3H, H_3_, H_4_, H_2_), 4.79 (s, 1H, H_1_), 3.91–3.36 (11H, H_6a_, H_6b_, H_5_, _Hg_, H_a_, H_a’_, H_f_), 1.74–1.69 (m, 4H, H_h_), 1.55 (4H, H_b_, H_e_), 1.39–1.25 (48H, H_c_, H_d_, H_i_-H_s_), 0.87 (t, 6H, J_t,s_ = 6.6Hz, Ht) (Figure S12); ^13^C NMR (75 MHz, CDCl_3_) δ 160.3 (C_amide_), 136.8 (C_Arom_), 105.2 (C_Arom_), 104.0 (C_Arom_), 97.6(C1), 69.7, 69.1, 68.4, 68.2, 66.2, 62.5, 31.9, 29.6, 29.66, 29.60, 29.5, 29.37, 29.35, 29.2, 26.0, 22.6, 14.1 (CH_3_) (Figure S13). ESI+-HRMS: *m/z* found [M+H]^+^: 808.6307, *m/z* calcd for C_47_H_85_NO_9_ [M+H]^+^ 808.6258.

*N-[O-(α-D-mannopyranosyl)oxyhexyl]-3.5-bis(hexadecyloxy)benzamide* (**25**). Prepared according to General Procedure **E** as a white solid (17 mg, 0.019 mmol, 89%).^1^H NMR (300 MHz, CDCl_3_) δ 6.87 (d, J_ortho,para_ = 2.1 Hz, 2H_ortho_), 6.53 (s, 2H, NH, H_para_), 4.97 (s, 2H, H_3_, H_4_), 4.81 (s, 2H, H_2_, H_1_), 4.38 (s, 1H, H_6a_), 3.92–3.38 (14H, H_6b_, H_5_, H_g_, H_a_, H_a’_, H_f_), 1.78–1.74 (m, 4H), 1.56–1.54 (m, 4H), 1.46–1.16 (m, 53H), 0.9–0.86 (t, J = 6.7 Hz, 6H) (Figure S14);^13^C NMR (75 MHz, CDCl_3_) δ 167.5(A_rC = O_), 160.2 (C_meta_), 136.6 (C_ipso-_), 105.4 (C_ortho_), 104.4 (C_para),_ 99.9, (C_1_), 72.3, 71.8, 68.2, 67.5, 66.4, 60.7, 39.9, 31.8, 29.6, 29.6, 29.5, 30.2, 28.1, 29.3, 29.3, 29.1, 26.5, 25.8, 22.63, 14.06 (Figure S15). HRMS**:** m/z found [M+H]^+^ 864.6930, m/z calcd for C_51_H_93_NO_9_ [M+H]^+^ 864.6923.

### 2.2. Biology

#### 2.2.1. Reagents

Dynantin was provided by the laboratory of Dr. PW Schiller and was prepared according to the previously published methods and stored at −20 °C as a lyophilized powder [24]. Purity of the peptide was determined to be ≥98% by RP-HPLC. Stock solutions of the peptide were generated in ddH_2_O (5 µg/µL), aliquoted and stored at −80 °C. The preparation of ML-C_12_ followed the previously published methods [22], with purity determined to be ≥95% as indicated by thin-layer chromatography and NMR spectroscopic analyses. Stock solutions (50 µg/µL) of **24** or ML-C_12_ were prepared by dissolving the compounds in THF (HPLC grade, Fisher Scientific, Fairlawn, NJ, USA), and stored at −20 °C. When required, less concentrated solutions were prepared by dilution of these stocks in THF. Stock solutions of Nile Red (Sigma, St-Louis, MO, USA) were prepared at 10 mg/mL in DMSO (Corning, Manassas, VA, USA).

#### 2.2.2. Human Plasma Collection

Blood was collected from healthy volunteers in EDTA blood collection tubes (BD Vacutainer, Mississauga Canada). Plasma was collected after centrifugation at 900× *g* and 20 °C for 10 min with decreased deceleration, aliquoted, and stored at −80 °C. All protocols were approved by the Research Ethics Board and the Biosafety Committee at Health Sciences North Research Institute (Protocol # 18-061).

#### 2.2.3. HPLC Conditions

All analyses were performed using a Shimadzu Prominence series HPLC system (Shimadzu Corporation, Kyoto, Japan), equipped with an LC-20AB binary pump (Serial: L20124200883), SIL-20A HT autosampler (Serial: L20345256104), CTO-20AC temperature-controlled column oven (Serial: L2021525077), SPD-M20A photodiode array detector and CBM-20A communications bus (Serial: L20235154327). All equipment were controlled by Shimadzu Lab Solutions Lite software version 5.71 SP2. For separation, an Ultra C18 column, 3 μm, 50 × 4.6 mm (RESTEK Corporation, Bellefonte, PA) was used. Dynantin samples were analyzed at a constant solvent flow rate of 0.7 mL/min at 35 °C using a binary gradient (Table 1). Solvent A consisted of a 25% solution of acetonitrile (HPLC grade, Fisher Scientific, Fairlawn, NJ, USA) in ddH_2_O (0.2 μm filtered) and solvent B consisted of acetonitrile with each solvent containing 0.1% trifluoroacetic acid (*v/v*, protein sequencing grade, Sigma Aldrich, Fairlawn, NJ, USA).

#### 2.2.4. Entrapment

Glycoliposomal entrapments were investigated according to previously published methods [23,25,26]. Samples were setup in triplicate, with the mixtures gently vortexed for 5 min. The degree of the Dynantin peptide entrapment was represented as the percentage of entrapped peptide relative to the amount determined in respective control samples comprised of the peptide and THF devoid of any glycolipid.

#### 2.2.5. Plasma Stability Analysis

Dynantin stability was also investigated according to previously published methods [23,25,26]. Samples were setup in triplicate, and stability is represented as the percentage of peptide remaining relative to the amount determined at T-zero.

#### 2.2.6. Evaluation of Hemolysis

Red blood cells (RBC) were isolated from the blood of three healthy volunteers by Ficoll-Paque (GE Healthcare, Uppsala, Sweden) density gradient separation (approved by the Research Ethics Board and the Biosafety Committee at Health Sciences North Research Institute, protocol # 18-061). Isolated RBC were diluted to 5% (*v/v*) in PBS containing various concentrations of **24** (12.5-100 μg/mL). PBS alone was used as a 0% hemolysis control and ACK lysing buffer (Lonza, Walkersville MD) was used as a 100% hemolysis control. Hemolysis was measured according to a previously published protocol [38]. RBC were platted in a 96 multi-well plate (200 μL) and incubated at 37 °C for 1 h, after which the plate was centrifuged at 300× *g* for 10 min. 100 μL of the supernatants was transferred to a new plate and absorbance was read at 570 nm with a Synergy H4 Hybrid Microplate Reader (BioTek, Winooski, VT, USA).

#### 2.2.7. Cell Culture and Nile Red Uptake

Murine dendritic cells JAWSII (ATCC^®^ CRL-11904™) were obtained from ATCC and grown in RPMI-1640 medium (HyClone, Logan, UT, USA) supplemented with 8% FBS (Gibco, Grand Island NY), 100 units/mL penicillin/streptomycin (HyClone, Logan, UT, USA) and 5 ng/mL of GM-CSF (Invitrogen, Frederick, MD, USA). Murine macrophage cells J774A.1 (ATCC^®^ TIB-67™) were obtained from ATCC and grown in Dulbecco’s minimal essential medium (DMEM) high glucose (HyClone, Logan, UT, USA) supplemented with 10% FBS and 100 units/mL penicillin/streptomycin. The human ovarian cancer cell line A2780 was provided by Dr. Barbara Vanderhyden (Ottawa Hospital Research Institute, Ottawa, ON, Canada) and the human colon cancer cell line HCT 116 (ATCC^®^ CCL-247™) was provided by Dr. Hoyun Lee (Health Sciences North Research Institute, Sudbury, ON, Canada). Both cancer cell lines were grown in RPMI-1640 supplemented with 10% FBS and 100 units/mL penicillin/streptomycin. All cells were grown at 37 °C and 5% CO_2_. When confluent, JAWSII, A2780 and HCT 116 were collected using 0.25% Trypsin EDTA (Gibco, Grand Island, NY, USA), and J774A.1 were collected using a cell scraper. Viability was assessed prior to experiments by Trypan Blue (Gibco, Grand Island, NY, USA) exclusion where the viability was >90%. Cells were seeded at 3 × 10^5^ cells in 300 μL in a 96 multi-well plate and left untreated or were treated with 10 μg/mL of empty **24** or ML-C_12_, 1 μg/mL of Nile Red (Sigma, St-Louis, MO, USA), or 1 μg/mL of Nile Red encapsulated in 10 μg/mL of **24** or ML-C_12_. Each formulation for treatment was prepared as a 50x stock (50 µg/mL Nile Red, 0.5 mg/mL liposome) in sterile ddH_2_O. Cells were collected after 1 h at 37 °C, washed once with 300 μL of PBS, and re-suspended in 300 μL of PBS. Mean fluorescence intensity (MFI) was analyzed using the Cytomics FC-500 flow cytometer (Beckman Coulter, Fullerton, CA, USA) using the FL3 channel where 10^4^ events were measured. Data was analyzed using the CXP Analysis Software. Nile Red in J774A.1 and JAWSII was also visualized using an inverted Olympus IX73 microscope and excited with the TRITC filter at 540 nm for 2 s or phase contrast for <59 ms and merged for the final image.

#### 2.2.8. Cytokine Analysis

The cytokine profile was assessed in peripheral blood mononuclear cells (PBMCs) isolated by Ficoll-Paque (GE Healthcare, Uppsala, Sweden) density gradient from the whole blood of three immunocompetent healthy individuals (in accordance to HSN REB #18-061). Immediately after extraction, PBMCs were seeded at 10^6^ cells/mL in RPMI-1640 medium (HyClone, Logan, UT, USA) supplemented with 10% FBS (Gibco, Grand Island NY) and 100 units/mL of penicillin/streptomycin (HyClone, Logan, UT, USA). PBMCs were left untreated or treated with **24** (10 μg/mL) for 48 h before analysing the supernatants for IL-12p40, IL-12p70, IL-10, IL-2, TNFα and IFNγ by ELISA. ELISA kits were purchased from R&D Systems (Minneapolis, MN, USA) and ran according to the manufacturer’s protocols.

#### 2.2.9. Statistical Analyses

Statistical analyses in the form of one way ANOVA with a Tukey HSD were performed using Graph Pad Prism 5.

## 3. Results & Discussion

### 3.1. Syntheses & Characterization of Neoglycolipids

#### 3.1.1. Synthesis of the Lipid Architecture

**Scheme 1:**
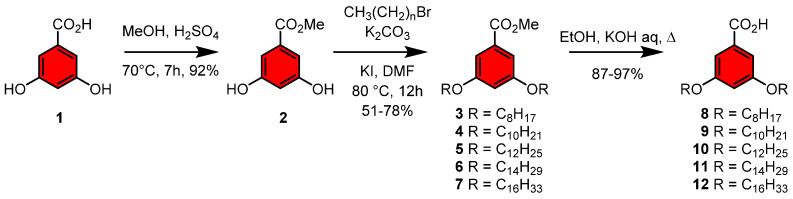
Syntheses of the lipid architecture using 3,5-dihydrobenzoic acid.

**Scheme 2:**
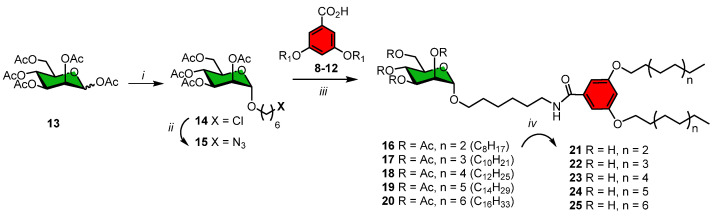
Syntheses of the mannosylated neoglycolipids using a one-pot modified Staudinger reaction between azide **15** and acids **8–12** with a hexyl side-chain at the sugar residue. Reagents and conditions: *(i)* Cl(CH_2_)_6_OH, Et_2_OBF_3_, DCM, 0 °C, 1h, then 40 °C, 18h (66%); (*ii)* NaN_3_, NaI, DMF, 24 h, 80 °C, (93%); (*iii)* Bu_3_P, DCM, HOBt, DIC, 24 h; (*iv)* NaOMe, MeOH, 3 h., quant.

The syntheses of the neoglycolipids were performed using a two stages convergent approach as described in Scheme 1 and Scheme 2 which involved the preparation of the lipid tails and the sugar moieties. First, the syntheses of the lipid architectures were based on a 3,5-dihydroxybenzoic acid scaffold **1 [30,32,33]**. Protection of the acid functionality as a methyl ester (MeOH, H_2_SO_4_) afforded **1** in 92% yield, followed by alkylation with C_8_-C_16_ alkyl bromides (RBr, KI, K_2_CO_3_, DMF, 80 °C) to provide intermediates **3**–**7** in 51-78% yields. This was followed by the methyl esters hydrolysis (KOH, EtOH) to give lipids **8**–**12** in 87-97% yields (Scheme 1). The procedure was analogous to the one previously published for related glycodendrimersomes [30,32,33].

#### 3.1.2. Synthesis of Mannosylated Neoglycolipids

The necessary mannopyranoside moiety was prepared from mannose pentaacetate **13** which was initially glycosidated with 6-chlorohexanol using BF_3_-OEt_2_ in DCM as Lewis acid catalyst to give the known chloride **14** in 66% yield (Scheme 2) [36,37] Substitution of the chloride by an azide functionality was also done following a known procedure (NaN_3_, NaI, DMF, 80 °C) to afford azide **15 [37]**. We chose a hexyl linker in the mannopyranoside residues since it afforded a potent ligand for a variety of mannose binding proteins [29]. The chemical ligation between the above lipid architectures **8**–**12** as acid moieties and the azide **15**, presenting a clean α-anomeric configuration, was done in a new one-pot modified Staudinger reaction (Bu_3_P, HOBt, DIC, DCM) in order to avoid *O*- to *N*-acyl shift typically observed when peracetylated sugars are simultaneously present with free amine if one would have used classical peptide coupling reaction. This approach gave rise to peracetylated neoglycolipids **16**–**20** in good to moderate yields that appear more practical to the one using glycosidation of preformed linker-tied lipids harboring alcohol head groups [39,40]. Uneventful *trans*-esterification under Zemplén conditions (NaOMe, MeOH) provided mannosylated liposome precursors **21**–**25** in yields ranging from 77-98%. Products were fully characterized by ^1^H- and ^13^C-NMR spectroscopy and by mass spectrometry. Importantly, beyond the advantages of the synthetic strategy present above, the novel mannosylated compounds **21**–**25** possessed a longer alkyl linker that may improve the availability of the sugar heads for binding to their associated receptor. In addition, as opposed to ML-C_12_, the new library of compounds were devoid of stereocenters.

#### 3.1.3. Mannosylated Neoglycolipid Self-Assembly Analysis by DLS and TEM

**Figure 2:**
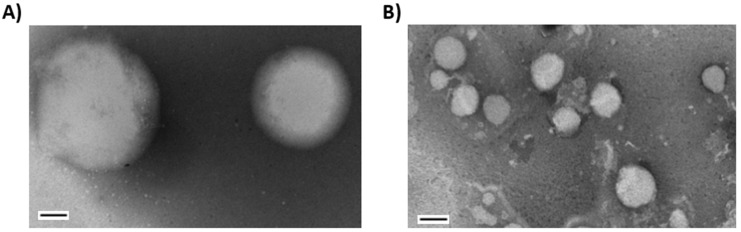
Transmission electron micrographs (TEM) of (**A**) ML-C_12_ and (**B**) **24**. Solutions of the glycolipids (0.5 µg/uL in ddH_2_O) were analyzed after incubation at room temperature for 24 h. The preparation was negatively stained with 3% phosphotungstic acid and imaging performed at 75 kV. Bar = 100 nm.

The simplest method for the self-assembly of amphiphilic molecules into vesicles and liposomes involves the injection of their solution in a water-miscible solvent such as ethanol or THF into water or buffers [41]. This methodology has been shown to be efficient for the self-assembly of previously described amphiphilic analog ML-C_12_, prepared by unprecedented thiol-yne ligation between sugar alkynes and thiols [22]. The method was also found suitable using stable amphiphilic Janus dendrimers into monodisperse vesicles called dendrimersomes, as previously described [30,32,33]. The resulting assemblies were first analyzed by dynamic light scattering (DLS) for size, polydispersity (PDI), and stability in time. These data are summarized in Table 2. The dimensions of all assemblies from Scheme 2 ranged from 252 to 385 nm which are suitable for drug delivery and other applications [42,43,44]. Mannolipid **25**, harboring a hexyl-linked mannoside and two C_16_ residues in the lipid tail was readily precipitating soon after their self-assembly into larger initial aggregates (766 nm). Thus, this unbalanced hydrophilic/hydrophobic combination was found unsuitable for the following experiments and was consequently abandoned. It was also noticed that compound **24**, having the hexyl linked-mannoside and two C_14_ lipid tails formed the most stable vesicles with proper size (287 nm) and the lowest polydispersity (0.04). Nanoparticle formation was further confirmed via TEM analysis, where ML-C_12_ and aromatic glycolipid **24** were compared (Figure 2). Interestingly, particle sizes observed with **24** were smaller than those with ML-C_12_**.** Compound **24** was therefore selected for other ongoing applications [25,26,45].

#### 3.1.4. Binding & Agglutination Abilities of Mannoliposomes with Con A

Mannosylated liposomes are of interest to enable specific multivalent glycoconjugate interactions with their cognate protein receptors in lectin-targeted delivery of a cargo onto cells [6,7,8,9,10,11,12,13,14,15,16,17,18,19,20,21], a scenario that has been extremely valuable in the inhibition of binding of Ebola viruses to dendritic cells by mannosylated NPs [46,47]. An essential prerequisite for considering pharmaceutical applications is to ascertain the ligand availability of the sugar head groups in molecular recognition experiments with carbohydrate-binding receptors (lectin). Agglutination of mannosylated glycoconjugates by lectins can clearly validate such multimodal binding interactions [31]. To demonstrate whether our mannosylated liposomes can be used for the targeting of cells, we first conducted experiments on their agglutination ability the plant lectin concanavalin A (Con A) from *Canavalia ensiformis* (Jack bean). Obviously, the process implies cross-linking.

Con A is a 104 kDa homotetrameric protein comprised of four identical subunits (26.5 kDa, 235 amino-acids) and exists as an active tetramer under physiological pH (*ex*. PBS/pH 7.4) [48]. Its optimal carbohydrate binding ligand is α-D-mannopyranoside with unmodified OH groups at C-3, C-4, and C-6 [49,50]. Con A agglutinates red blood cells (RBCs) and interacts with immunoglobulin’s glycans [51]. It binds bacteria and viruses with exposed mannose residues [52]. Mannopyranosides-containing nanoparticulate conjugates are commonly used to target dendritic cells through their DC-SIGN receptors [53,54]. For this reason, they are often used in vaccine formulations [55]. DC-SIGN (Dendritic Cell-Specific Intercellular adhesion molecule-3-Grabbing Non-integrin) also known as CD209 (Cluster of Differentiation 209) is a C-type lectin receptor present on the surface of both macrophages and dendritic cells [53,54,55]. It binds avidly to mannopyranosides of glycoproteins which are abundantly expressed on cell surfaces and on pathogenic agents, with viruses being well documented examples. These receptors are ubiquitously distributed on cancer cell as well.

**Figure 3:**
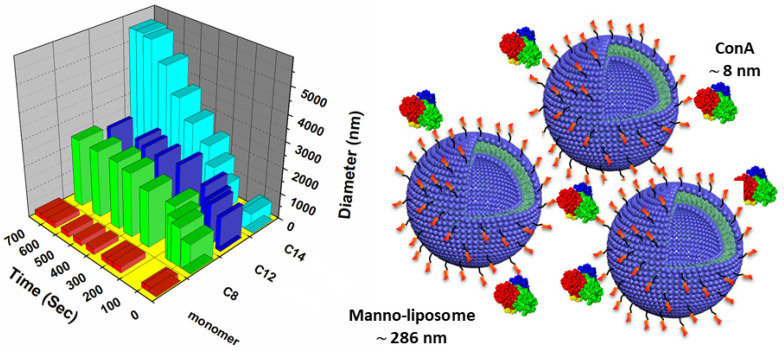
Time scale agglutination of mannosylated liposomes **21** (C_8_), **23** (C_12_), and **24** (C_14_) in the presence of Con A (4 binding sites/mol) at 0.5 mg/mL (100 μL) in PBS.

As seen in Figure 3, the ability of our preformed hexyl-bearing mannosylated neoglycoliposomes **21** (C_8_), **23** (C_12_), and **24** (C_14_) could rapidly form larger aggregates in the presence of the tetrameric lectin ConA. The size of the aggregates was followed as a function of time by DLS and the results clearly showed that within ~10 min., compound **24** had the best performance.

In comparing the results of the agglutination studies with those previously performed with ML-C_12_ [22], the agglutination of particles formed with **24** occurs much more rapidly and to a greater extent than those formed with ML-C_12_. This suggests that the mannose residue in **24** is more freely available for protein binding, confirming our hypothesis about an inadequate distance between the sugar head group and lipid tails outlined earlier. In fact, the enhanced agglutination of liposomes assembled from **24** occurred under a Con A concentration approximately 4X lower than that with ML-C_12_, further supporting that the additional alkyl linker between the mannose residue and the aromatic lipid architecture in **24** translates into glycoliposomes with a more freely available sugar group.

### 3.2. Biological Evaluation

Following the chemical characterization of the novel liposomes, we aimed to compare the biological activity of the new lead compound **24** to the previously characterized ML-C_12_ by assessing lipophilic drug uptake and preliminary toxicity in a variety of cells, followed by evaluation of entrapment efficacy and stabilization potential using Dynantin as a model. The biological evaluation specifically aimed to complement our previous in vitro and in vivo work that compared both **24** and ML-C_12_ in various liposomal formulations containing cholesterol [25,26,45].

#### 3.2.1. Cellular Uptake of Mannosylated Liposomes

**Figure 4:**
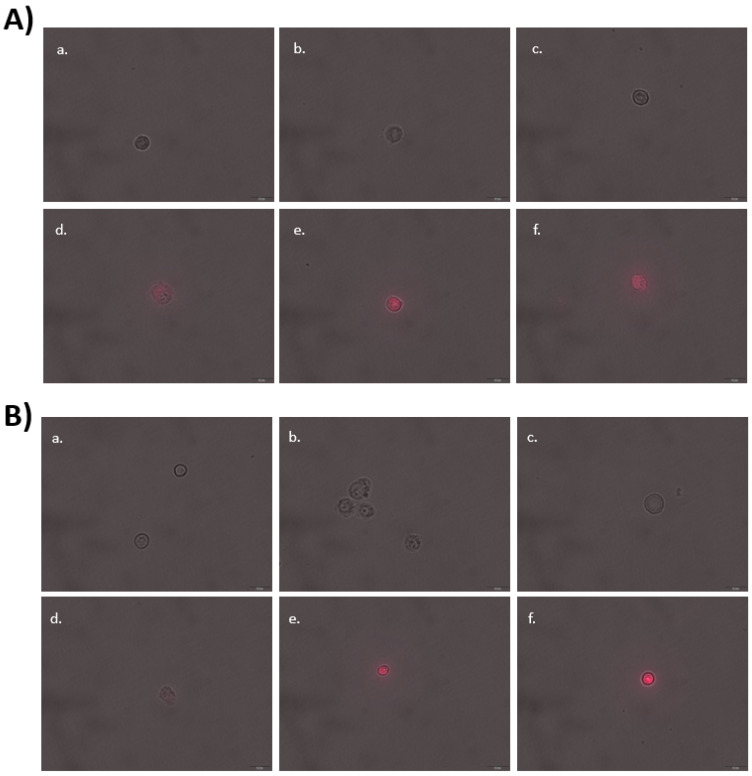
Visualization of Nile Red with and without **24** or ML-C_12_ in (**A**) JAWSII and (**B**) J774A.1. Cells were treated for 1 h without (**a**–**c**) or with (**d**–**f**) Nile Red. Images were obtained using an inverted Olympus IX73 microscope and are merged with phase contrast and TRITC, 400× magnification. Cells were exposed at 540 nm for 2s. Scale bar, 20 µm. (**a**) negative control; (**b**) ML-C_12_; (**c**) **24**; (**d**) negative control with Nile red, (**e**). ML-C_12_ with Nile Red; (**f**) **24** with Nile Red.

**Figure 5:**
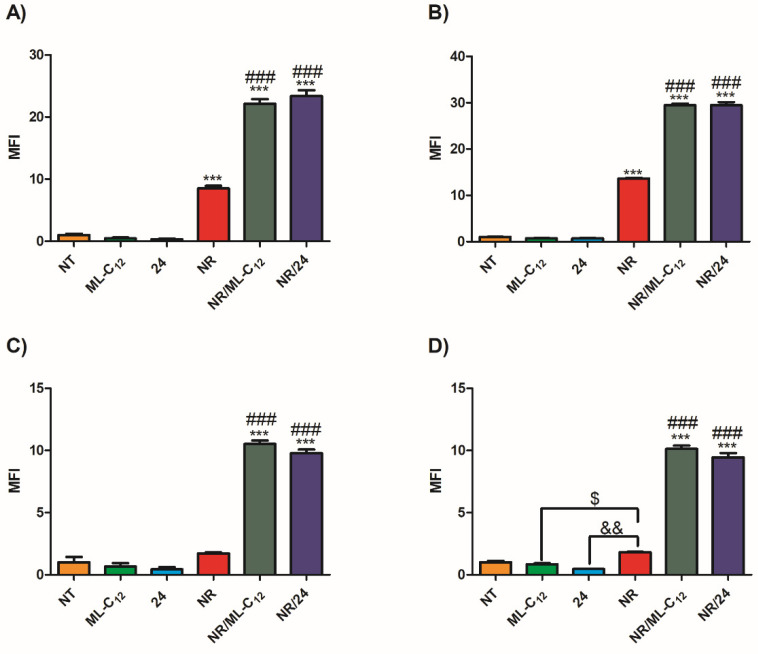
Nile red (NR) uptake by (**A**) JAWSII, (**B**) J774A.1, (**C**) HCT 116, (**D**) A2780 cells with ML-C_12_ or **24.** Cells were seeded at 3 × 10^5^ cells in 300 μL in a 96 multi-well plate and were left untreated (NT) or were treated with 10 μg/mL of empty ML-C_12_ or **24**, 1 μg/mL of Nile Red (NR), or 1 μg/mL of Nile Red encapsulated in 10 μg/mL of liposome (ML-C_12_ or **24**). Cells were collected after 1 h at 37 °C, washed once with 300 μL of PBS, and suspended in 300 μL of PBS. Mean fluorescence intensity (MFI) of singlets was measured by flow cytometry (10^4^ events). Data is normalized to the NT control (MFI = 1). Statistical differences were measured using a one-way ANOVA with a Tukey’s HSD where *** *p* < 0.001 as compared to NT, ML-C_12_ and **24**, ^$^ *p* < 0.05 as compared to ML-C_12_, ^&&^ *p* < 0.01 as compared to **24,** and ^###^ *p* < 0.001 as compared to NR alone. Data are shown as the average ± SEM of a triplicate from one experiment.

Studies of the behavior of the mannosylated glycoliposomes in biological systems were performed in various cell types representative of the targeting potential of the mannose receptor [8,9,10,11,12,13,14,15]. The murine JAWSII dendritic cell and J774A.1 macrophage cell lines were chosen to represent immunological cell targeting [8,9,10,11,12,13,14], and by extension, the macrophages and microglia of the blood–brain barrier (BBB) [16]. The binding of particles formed with either **24** or the alkyl-bearing glycolipid ML-C_12_ in JAWSII and J7774A.1 cells after encapsulation of the hydrophobic dye Nile Red using fluorescence microscopy and flow cytometry (Figure 4) were first compared. Fluorescent Nile Red can be used as a probe to measure intracellular delivery of hydrophobic drugs using nanoparticles [56]. Here, particle binding was increased by mannosylation, which was evidenced by the increased cellular fluorescence observed qualitatively under the microscope, and confirmed quantitatively by the cytometric analysis (Figure 5). The flow cytometric analysis of Nile Red uptake in cancer cells using the colon cancer cell line HCT 116 and the ovarian cancer cell line A2780 showed similar results (Figure 5). In all cases, there was no statistical difference in the targeting ability between **24** and the alkyl bearing glycolipid ML-C_12_, thus inferring on the critical role played by mannoside receptors for the uptake of our mannosylated liposomes.

While the results presented above clearly demonstrate that both mannosylated liposomes ML-C_12_ and **24** can be used to enhance uptake of hydrophobic drugs in immune and cancer cells, correlating to the apparent availability of the mannoside residues, it is worth noting that the experimental conditions are far from being optimized. It is possible that the glycolipid concentrations used could be leading to saturation with respect to available mannose residues, thus masking any differences in the targeting capacity of the respective glycoliposomes. Moreover, in vitro experiments such as these are not predictive of in vivo behavior, where other pharmacokinetic aspects such as biodistribution and stability will be at play. In fact, in our investigations of the CNS targeting ability of optimized glycoliposomes formulations containing ML-C_12_ or **24** and cholesterol, the results were trending toward an improved distribution of Dynantin into the brain for the aromatic-containing particles [26]. The optimized formulation for **24** contained a lower molar ratio of glycolipid than that for ML-C_12_, which suggests an enhanced targeting capability for particles assembled from the aromatic containing glycolipid, and correlates with the results of the agglutination studies presented herein which demonstrate a more freely available mannose residue in particles containing **24**. Again, while our in vivo studies were only preliminary and lacked optimization in terms of dosing and route of administration, we ultimately believe that glycoliposomes assembled from **24** will prove better for in vivo targeting and distribution.

Overall, the results demonstrate that glycoliposomes assembled from either ML-C_12_ or **24** enhance payload uptake in a variety of different cell types with expressed varied sugar receptors such as CD206 or glucose transporters (GLUT), also known to bind mannosides [57]. The liposomal targeting of these cell types suggests potential applications in cancer treatment, both for the direct delivery of drugs to the cancer cells but also to target CD206-overexpressing M2-like tumor-associated macrophages present in the tumor microenvironment [58]. The targeting of immune cells, particularly dendritic cells which are key antigen-presenting cells, also offer an interesting avenue for the delivery of antigens, with applications both in vaccine development and cancer immunotherapy [59,60,61]. Corroborating the above statement, a formulation containing compound 24 and an influenza vaccine antigen was shown to induce a better protection in the form of both antibodies and cellular-based responses in an elderly mouse model, as compared to the antigen alone [45]. Interestingly, macrophages and microglia overexpressing CD206 are also found in the brain and may contribute to enhanced drug targeting for the treatment of a broad range of diseases affecting the CNS [61,62,63].

#### 3.2.2. Preliminary Safety Assessments of Mannosylated Glycoliposomes

**Figure 6:**
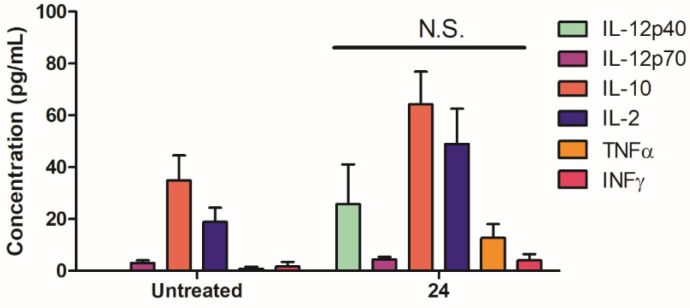
Immunogenicity assessment mannosylated liposomes assembled with **24.** Immune responses in PBMCs (isolated from 3 healthy volunteers) were measured. PBMCs were seeded at 10^6^ cells/mL and left untreated or treated with **24** (10 μg/mL). Cells were incubated for 48 h and supernatants were analyzed for the production of cytokines by ELISA. Statistical analysis in the form of an unpaired T-test was performed using GraphPad Prism and no significance (N.S.) was observed between the untreated and **24** treated groups for each respective cytokine.

Interaction with the immune system is a major consideration for liposomal delivery system development [64]. While favorable interaction with the immune system may be beneficial for the development of delivery systems in some cases, such as for vaccine development and immunotherapy [45,65], a liposome that is devoid of immune activity, or one that reduces inflammation, is necessary for other applications including CNS drug delivery and treatment of autoimmune diseases [66,67]. Our data demonstrated that glycolipid **24** did not significantly alter the cytokine profile in PBMCs isolated from healthy donors (Figure 6), and it had high hemocompatibility up to 100 µg/mL based on the lack of hemolysis following treatment of red blood cells (RBCs) (Table 3). Combined with our previously published findings, that showed that compound **24** was safe in mice at a dose of 50 µg/mouse (approximately 2.5 mg/kg), ans had negligible in vitro toxicity on PBMCs at a dose of 10 µg/mL [45], this data also strongly supports that compound **24** is suitable to develop various liposomal formulations targeted at a wide range of applications, including drug delivery into the brain [25,26] and cancer immunotherapy [45].

#### 3.2.3. Mannosylated Liposomal Entrapment of Dynantin & Protection against Plasma Degradation

**Figure 7:**
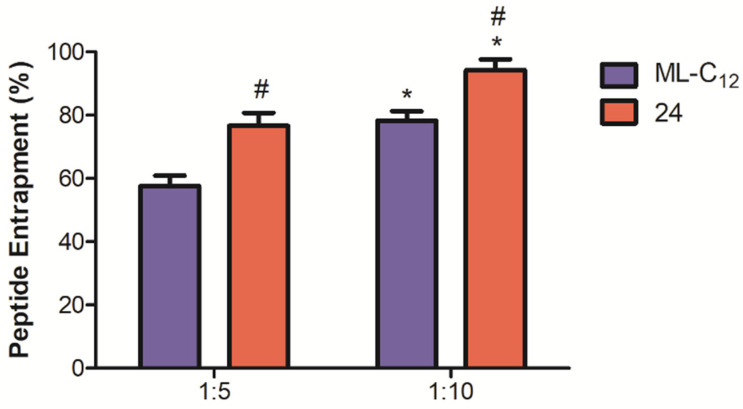
Glycoliposomal entrapment of Dynantin. Dynantin was combined with **24** or ML-C_12_ in water at 1:5 and 1:10 ratios of peptide: glycolipid. The degree of peptide entrapment was analyzed by RP-HPLC in the presence of 0.1% trifluoroacetic acid. The peptide was detected by absorbance at 210 nm and results are represented as the percentage of entrapped peptide relative the amount determined in control samples devoid of glycolipid. Data are shown as the average ± SEM of three separate experiments. ^#^ *p* < 0.05 as compared to ML-C_12_ for respective formulation, * *p* < 0.05 as compared to respective 1:5 formulation.

Similar to our previous works [23,25,26], the peptide KOR antagonist Dynantin [24] was used as a model payload to compare the ability of glycoliposomes assembled from **24** or ML-C_12_ to entrap and combat proteolytic degradation in human plasma ex vivo. Peptide entrapment levels with two different peptide: glycolipid ratios (1:5 & 1:10, *w/w*) were assessed (Figure 7). Entrapment levels with both **24** and ML-C_12_ glycoliposomes were higher with the 1:10 peptide: glycolipid ratio (94 ± 3% for **24**, 78 ± 2% for ML-C_12_) than the 1:5 ratio (77 ± 4% for **24**, 58 ± 3% for ML-C_12_). Overall, entrapment levels were statistically higher at either peptide: glycolipid ratios with particles formed with the novel aromatic bearing neoglycolipid **24** than our previous alkyl bearing glycolipid ML-C_12_. This suggest that glycoliposomes assembled from **24** are more ideal than those assembled from ML-C_12_ with respect to delivery systems applications where high levels of entrapment of therapeutic payloads are ultimately required.

**Figure 8:**
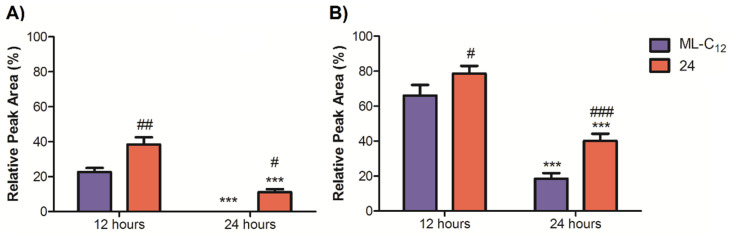
Plasma stability of glycoliposomal entrapped Dynantin. Dynantin was combined with **24** or ML-C_12_ at 1:5 (**A**) and 1:10 (**B**) ratios of peptide: glycolipid. Particles were incubated in human plasma at 37 °C and the levels of Dynantin remaining at various time points were analyzed by RP-HPLC in the presence of 0.1% trifluoroacetic acid and detected by absorbance at 210 nm. Peptide amounts were calculated relative to the quantities determined at time point zero, and data are shown as the average ± SEM of three separate experiments. *** *p* < 0.001 as compared to respective formulation at 12 h time point, ^###^ *p* < 0.001, ^##^ *p* < 0.01 and ^#^ *p* < 0.05 as compared to ML-C_12_ formulation at respective time point.

Dynantin is subject to rapid proteolytic hydrolysis in plasma, that which we have previously determined occurs in less than 12 h in our ex vivo plasma stability model system [23]. Plasma stability of the entrapped peptide was tracked over 24 h for either glycolipid at the two different peptide: glycolipid ratios (Figure 8). Particles formed with either glycolipid at the 1:5 peptide: glycolipid ratio extended protection beyond the 12 h time point (38 ± 35 remaining for **24**, 23 ± 2% remaining for ML-C_12_), while only particles formed with **24** were able to extend protection beyond the 24 h time point (11 ± 1% remaining). Similar trends were observed with the 1:10 peptide: glycolipid ratio, however the higher overall entrapment levels translated into higher peptide levels remaining at both the 12 h (79 ± 3% for **24**, 66 ± 5% for ML-C_12_) and 24 h time points (40 ± 3% for **24**, 18 ± 2% for ML-C_12_). Ultimately, liposomes formed with the aromatic bearing glycolipid **24** provided statistically better protection of Dynantin at either peptide: glycolipid ratio and time point than ML-C_12_, suggesting that particles formed with **24** are more resistant to degradation. These results are similar to those seen in previous work, where the addition of cholesterol resulted in particle formulations with enhanced stability for both **24** and ML-C_12_, but where formulations containing the aromatic glycolipid proved to be better [26]. Importantly, our previous work also confirmed that using ML-C_12_ or **24** in a liposomal formulation of Dynantin resulted in better brain delivery of the drug [25,26], with compound **24** trending towards having better delivery potential [26], corroborating the results shown herein.

## 4. Conclusions

This work successfully reports the chemical syntheses of a series of novel neoglycolipids having an exposed α-D-mannopyranoside residue and two lipid tails of varied alkyl chain lengths that are attached to an aromatic scaffold (Scheme 1 and Scheme 2). The work aimed to compare those novel mannosylated liposomal delivery systems to the previous formulations based on the shorter neoglycolipid ML-C_12_**.** The synthetic approach also aimed to address previous limitations, namely the generation of stereocenters within the lipid architecture, and inadequate spacing between the sugar and lipid moieties. An additional alkyl linker was included between the sugar moiety and the scaffold as it is generally well known that mannopyranoside receptors bind better when the sugar moiety possesses hydrophobic aglycones [29]. The overall synthetic strategy involved an improved procedure over the previous data [39,40] by using a modified Staudinger ligation of pre-formed, anomerically pure sugar azide-linker and carboxylic acid-ending lipid architectures, a modular approach that proved beneficial for the generation of libraries of neoglycolipids.

Self-assembly of the aromatic-bearing neoglycolipids into NPs of appropriate size was confirmed by DLS (Table 2) and TEM analyses (Figure 2). Overall, liposomes assembled from aromatic glycolipid **24** (C_14_ lipid chain length) showed ideal properties, and were evaluated further.

Glycoliposomes assembled from **24** demonstrated improved cross-linking ability in agglutination studies with the plant lectin Con A (Figure 3), indicating that the additional alkyl linker incorporated translates into NPs with a sugar residue that was more freely available for protein/receptor binding. While preliminary in vitro cellular uptake assays could not correlate this improved sugar availability with an enhanced targeting ability (Figure 4 and Figure 5), subsequent studies suggest that with optimization this might be realized in vivo [25,26]. Liposomes assembled from glycolipid **24** showed improved entrapment (Figure 7) and ability to protect a model peptide payload from degradation ex vivo in plasma (Figure 8). Finally, glycolipid **24** did not show any red blood cell toxicity, where various doses showed 0% hemolysis (Table 3), and respective glycoliposomes were non immunogenic when administered to PBMCs ex vivo (Figure 6), an important consideration for NP delivery systems.

Overall, the results highlight that the improved mannosylated neoglycoliposomes assembled from **24** described herein possess better biophysical properties than the previously described ML-C_12_ based particles [22,23]. In summary, **24** had a superior synthetic strategy, was devoid of chiral centers, had a longer alkyl linker that led to better sugar availability, and had superior entrapment and stabilization efficacy. These results conformed to our previous in vitro and in vivo findings [25,26,45]. The simplified architectures exemplified in this work appear to be more practical for several biological applications yet in development when compared to more elaborated glycodendrimersomes having multiple sugar head groups and multiple lipid tails. Beyond the applications in CNS diseases [25,26], works in progress are inclusively targeting other diseases such as cancer [45], inflammatory diseases, anti-infectious agents, as well as RNA delivery. Advantageously, the new synthetic strategy described herein can easily be used to modify the linker between the sugar head and lipid tails. Work is in progress to further expand our library of compounds using ether linker as opposed to alkyl linkers, allowing for optimization of the liposome rigidity. Finally, while the goal of the current work was to compare **24** to the previously described ML-C_12_, future work will aim to compare our characterized library of mannosylated compounds to commercially available liposomal delivery systems.

## Figures and Tables

**Table 1:** Solvent gradient program for the analysis of Dynantin entrapment, plasma stability and in vivo distribution using 25% acetonitrile in water (A) and acetonitrile (B), both with 0.1% trifluoroacetic acid (*v/v*).

Time (min)	Solvent	
	A (%)	B (%)
0	100	0
15	40	60
18	20	80
26	20	80
30	100	0
40	100	0

**Table 2:** Mannosylated neoglycolipid self-assembly analysis by DLS.

Compounds	Lipid Tail Size	Diameter (nm)	PDI
**21**	C_8_	252 ± 79	0.18
**22**	C_10_	n.d.	n.d.
**23**	C_12_	254 ± 41	0.20
**24**	C_14_	287 ± 27	0.04
**25**	C_16_	385 ± 20	0.91

n.d.: not determined due to precipitation issues.

**Table 3:** Hemolytic assay using different concentrations of mannosylated liposomes assembled from **24.** Red blood cells were isolated from three healthy volunteers and ran in triplicate with various concentrations of **24**. PBS was used as a negative control (0% hemolysis) and ACK lysing buffer was used as a positive control (100% hemolysis). All tested concentrations of **24** had 0% of hemolysis.

Compound	Concentration (μg/mL)	% Hemolysis (±SEM)
**24**	100	0
	50	0
	25	0
	12.5	0
PBS	-	0
ACK lysing buffer	-	100 ± 3

## Data Availability

All data is contained in the manuscript and accompanying supplemental materials.

## Supplementary Materials

The following supporting information can be downloaded at: https://www.mdpi.com/article/10.3390/pharmaceutics14112300/s1, Figure S1: 1H-NMR (300 MHz, CDCl3) of compound **16**, Figure S2: 1H-NMR (300 MHz, CDCl3) of compound **17**, Figure S3: 13C-NMR (75 MHz, CDCl3) of compound **17**, Figure S4: 1H-NMR (300 MHz, CDCl3) of compound **18**, Figure S5: 1H-NMR and 13C-NMR (300 and 75 MHz, CDCl3) of compound **19**, Figure S6: 1H-NMR (300 MHz, CDCl3) of compound **20**, Figure S7: 13C-NMR (75MHz, CDCl3) of compound **20**, Figure S8: 1H-NMR and 13C-NMR (300 and 75 MHz, CDCl3) of compound **21**, Figure S9: 1H-NMR-NMR (300 MHz, CDCl3) of compound **22**, Figure S10:13C-NMR (75 MHz, CDCl3) of compound **22**, Figure S11: 1H-NMR and 13C-NMR (75 MHz, CDCl3) of compound **23**, Figure S12: 1H-NMR (300 MHz, CDCl3) of compound **24**, Figure S13: 13C-NMR (300 MHz, CDCl3) of compound **24**, Figure S14: 1H-NMR (300 MHz, CDCl3) of compound **25**, Figure S15: 13C-NMR (75 MHz, CDCl3) of compound **25**.
