# Supplementary material for: Synthesis & Evaluation of Novel Mannosylated Neoglycolipids for Liposomal Delivery System Applications
Source: Pharmaceutics. 2022 Oct 26;14(11):2300. doi: 10.3390/pharmaceutics14112300 (PMC9692915; doi:10.3390/pharmaceutics14112300)
Supplement: Supplementary file 1 [file pharmaceutics-14-02300-s001.zip › pharmaceutics-1943752-supplementary.pdf]

Supplementary Information

Synthesis & Evaluation of Novel Mannosylated Neoglycolipids for Liposomal Delivery System Applications

Leila Mousavifar [1,†], Jordan D. Lewicky [2,†], Alexis Taponard [1], Rahul Bagul [1], Madleen Rivat [1], Shuay Abdullayev [1], Alexandrine L. Martel [2], Nya L. Fraleigh [2], Arnaldo Nakamura [3], Frédéric J. Veyrier [3], Hoang-Thanh Le [2,4,*] and René Roy [1,*]

1 Glycosciences and Nanomaterial Laboratory, Université du Québec à Montréal, P.O. Box 8888, Succ. Centre-Ville, Montréal, QC H3C 3P8, Canada
2 Health Sciences North Research Institute, 56 Walford Road, Sudbury, ON P3E 2H2, Canada
3 Armand-Frappier Santé Biotechnologie Research Centre, Institut National de la Recherche Scientifique, 531 Boulevard des Prairies, Laval, QC H7V 1B7, Canada
4 Medicinal Sciences Division, NOSM University, 935 Ramsey Lake Road, Sudbury, ON P3E 2C6, Canada
* Correspondence: hle@hsnsudbury.ca or hle@hsnri.ca (H.-T.L.); roy.rene@uqam.ca (R.R.)
† These authors contributed equally to this work.

NMR Spectra:

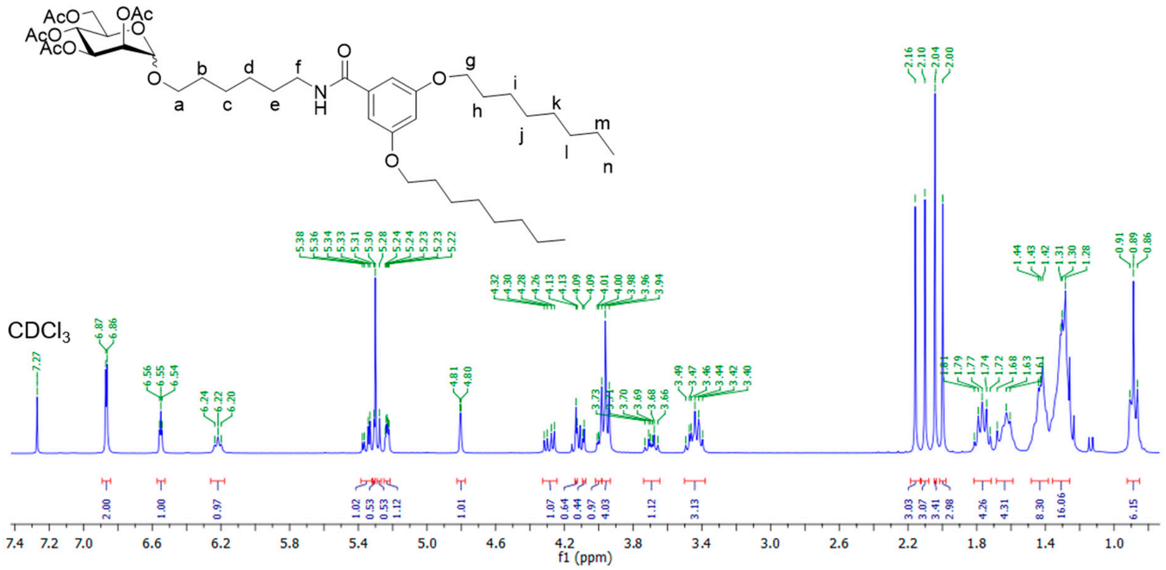

Figure S1. ^{1}H-NMR (300 MHz, $CDCl_3$) of compound **16**.

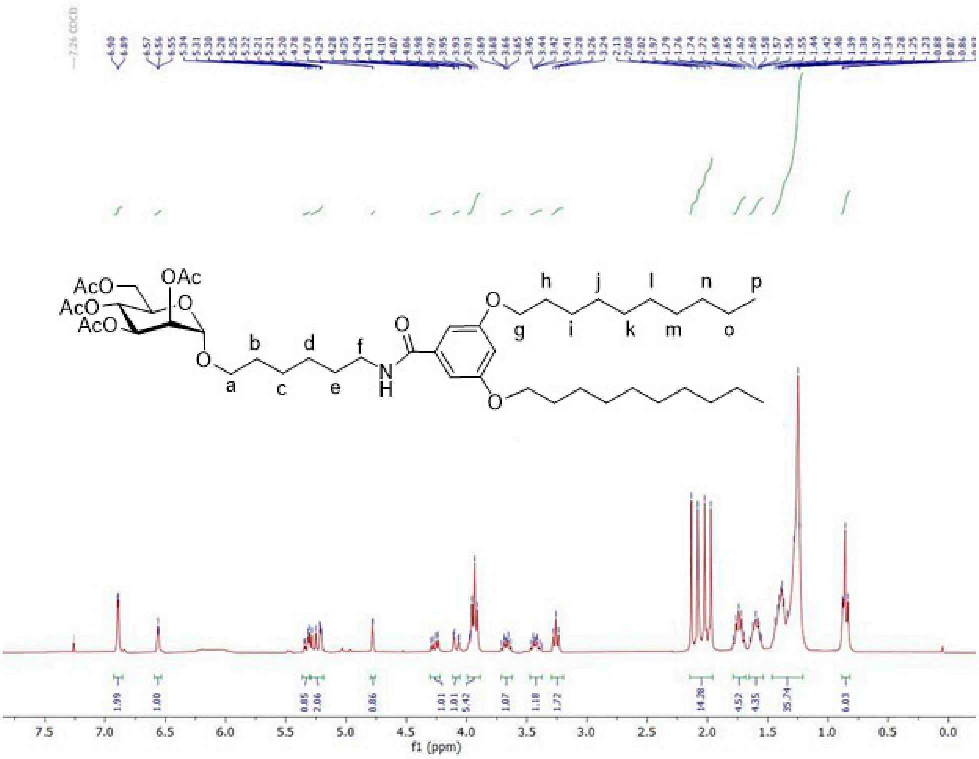

Figure S2. ^{1}H-NMR (300 MHz, $CDCl_3$) of compound **17**.

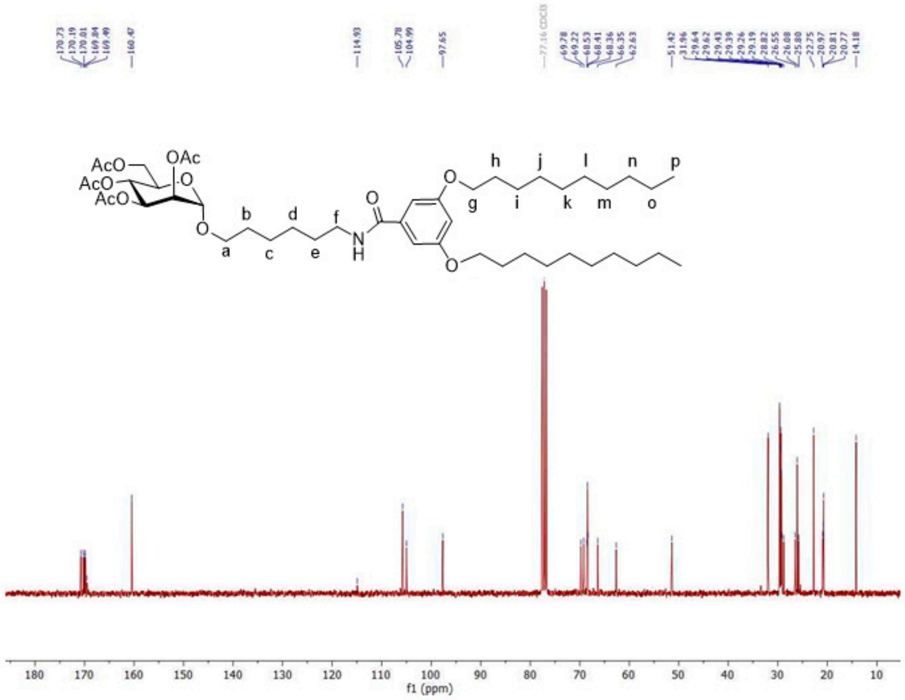

Figure S3. ^{13}C-NMR (75 MHz, $CDCl_3$) of compound **17**.

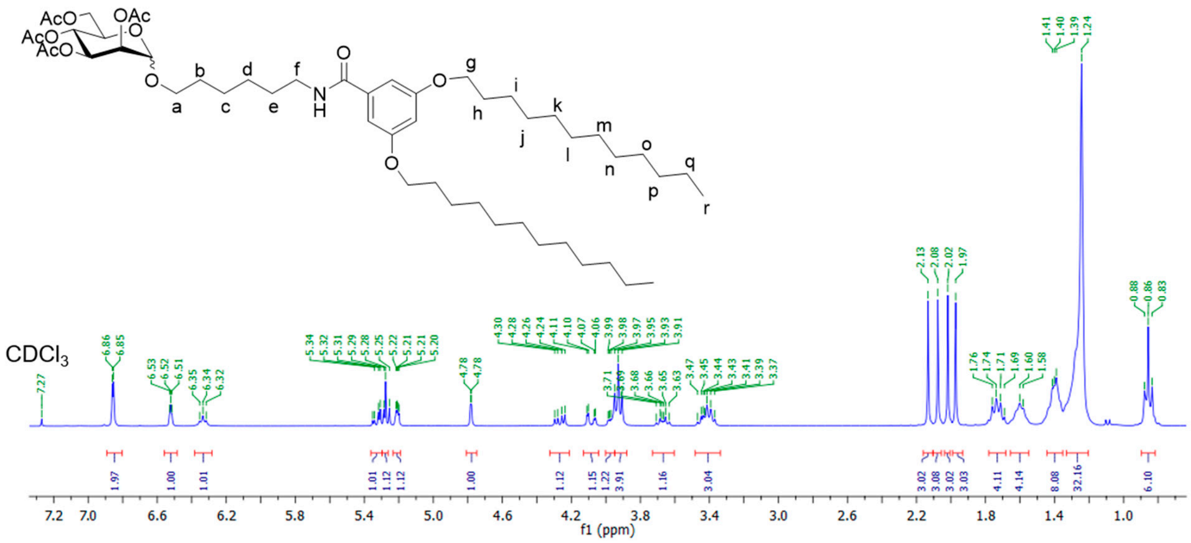

Figure S4. ^{1}H-NMR (300 MHz, $CDCl_3$) of compound **18**.

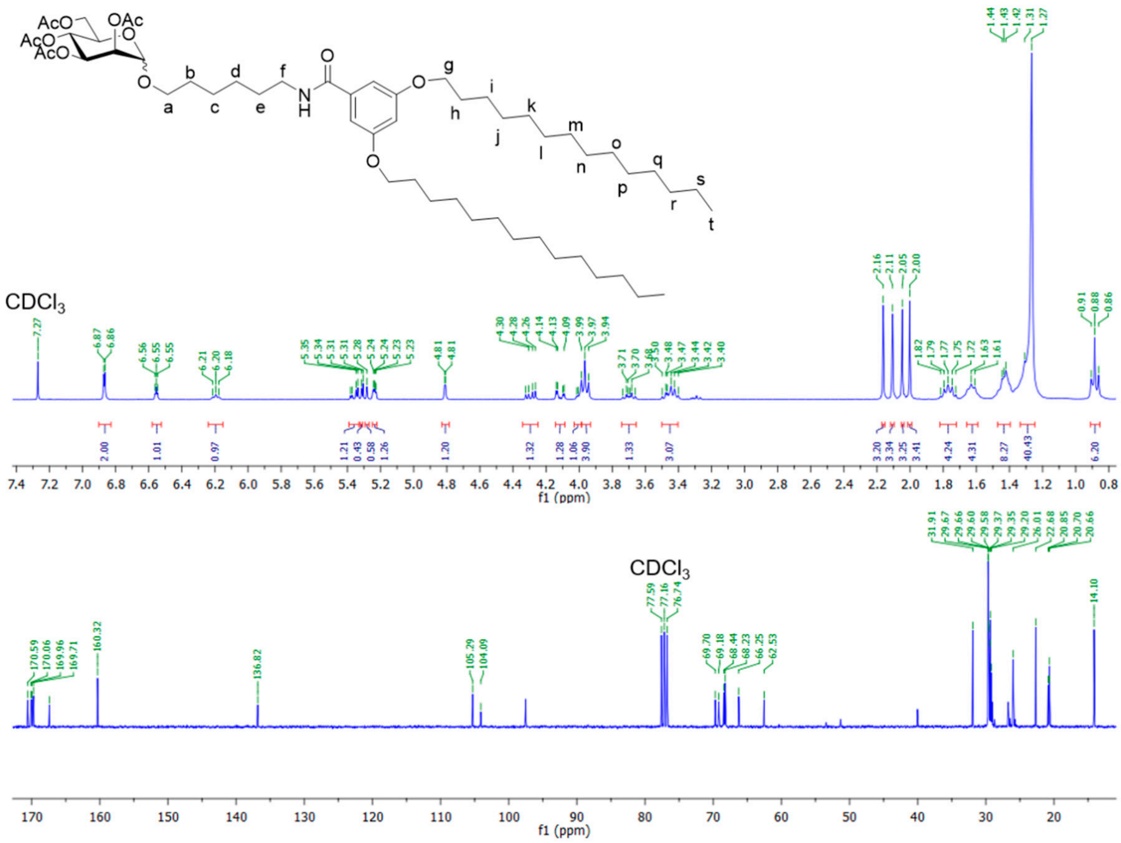

Figure S5. ^{1}H-NMR and ^{13}C-NMR (300 and 75 MHz, $CDCl_3$) of compound **19**.

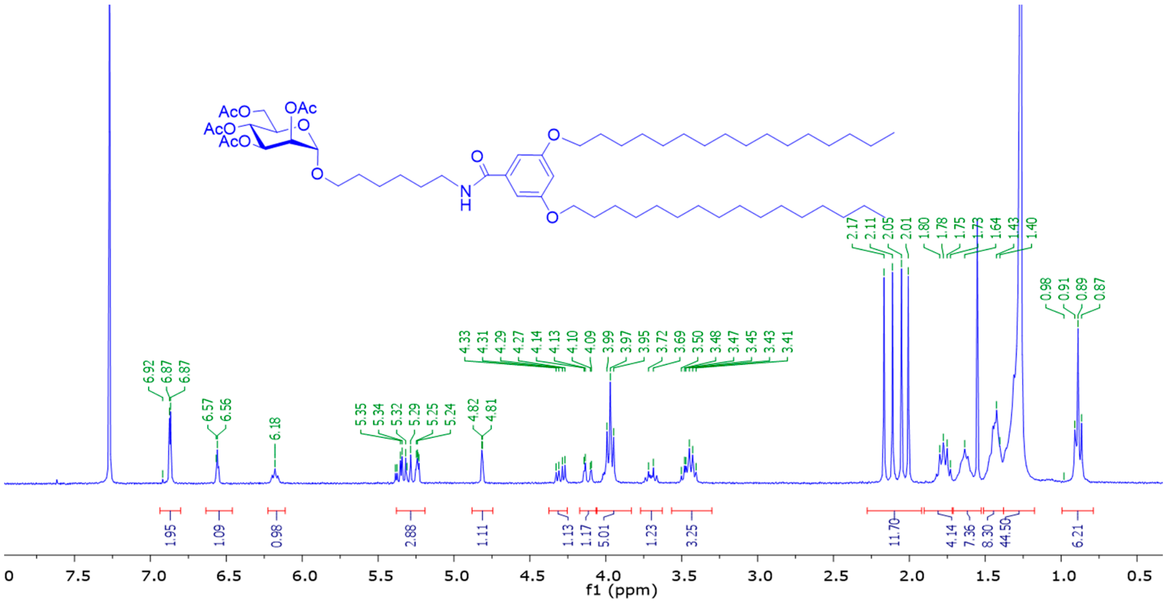

Figure S6. ^{1}H-NMR (300 MHz, $CDCl_3$) of compound **20**.

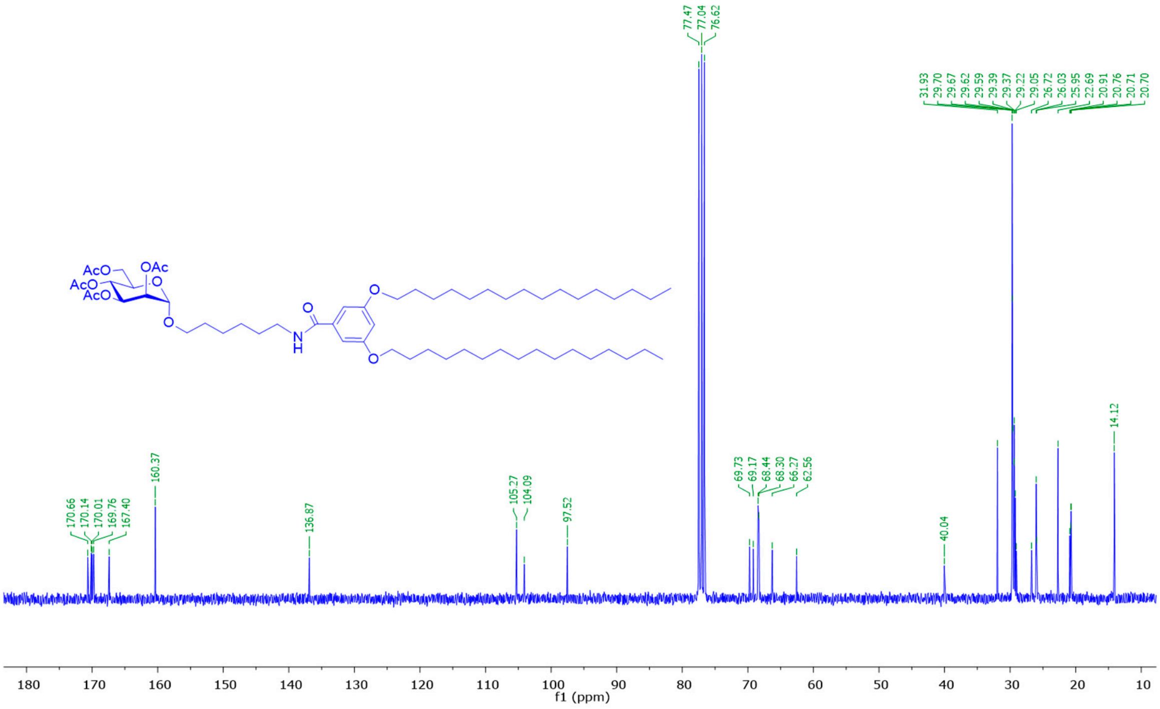

Figure S7. ^{13}C-NMR (75MHz, $CDCl_3$) of compound **20**.

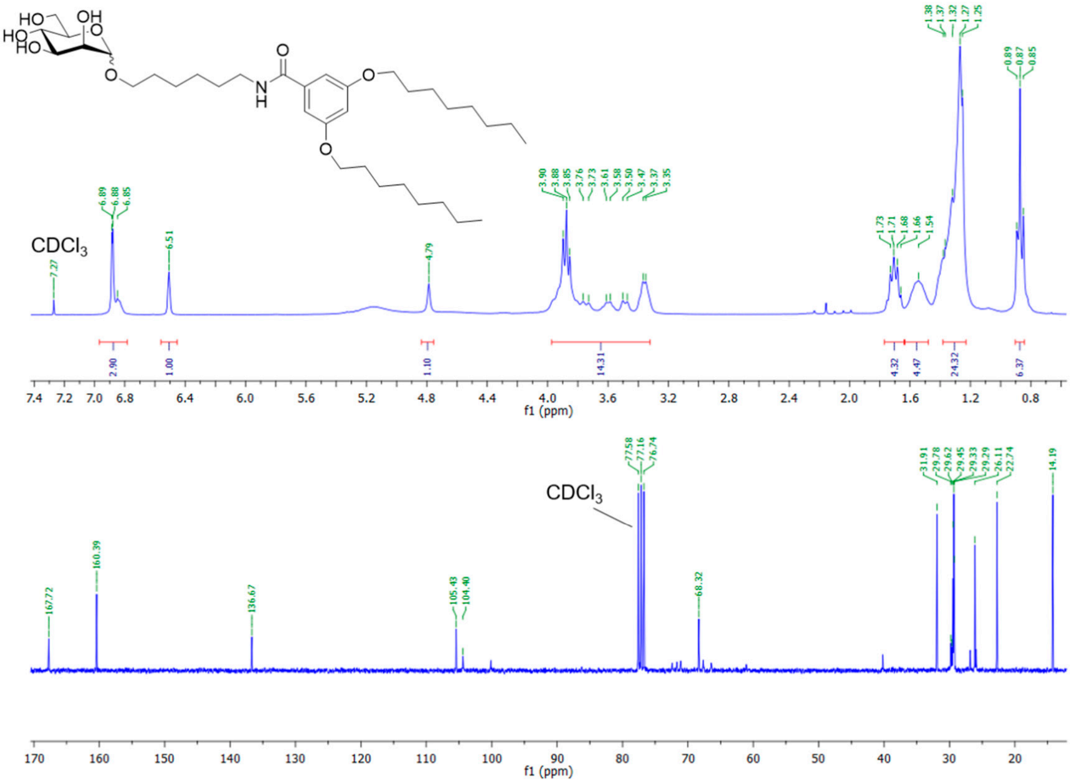

Figure S8. ^{1}H-NMR and ^{13}C-NMR (300 and 75 MHz, $CDCl_3$) of compound **21**.

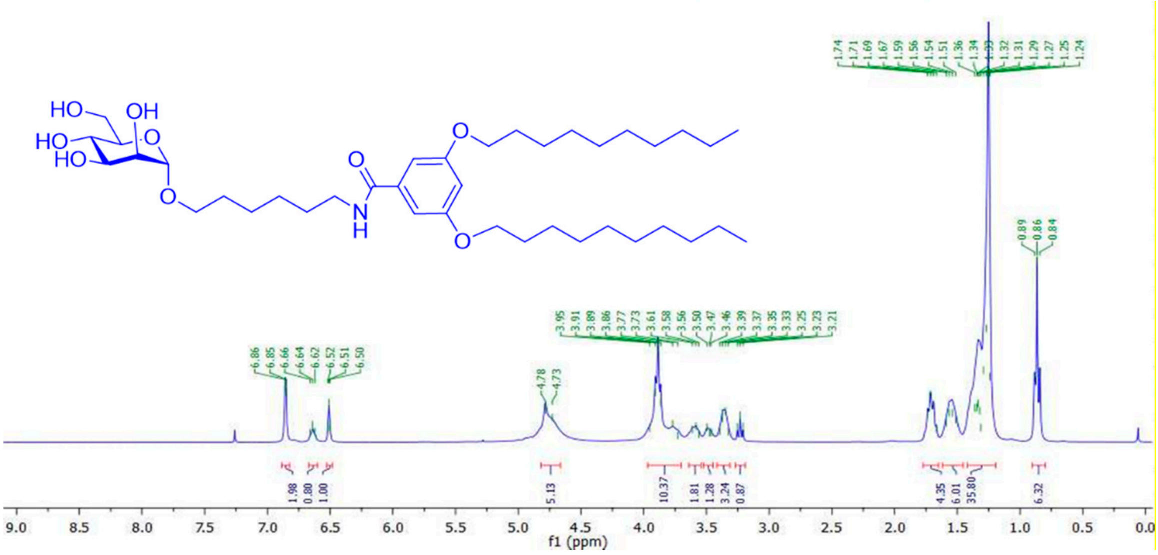

Figure S9. ^{1}H-NMR-NMR (300 MHz, $CDCl_3$) of compound **22**.

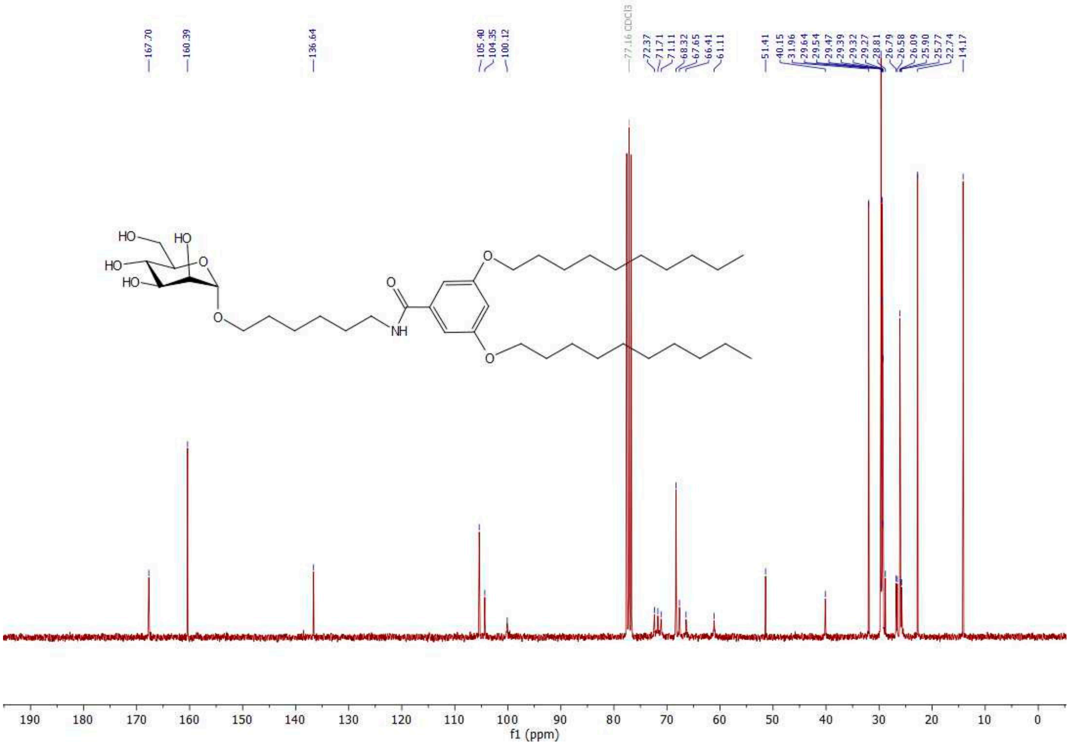

Figure S10. ^{13}C-NMR (75 MHz, CDCl$_3$) of compound **22**.

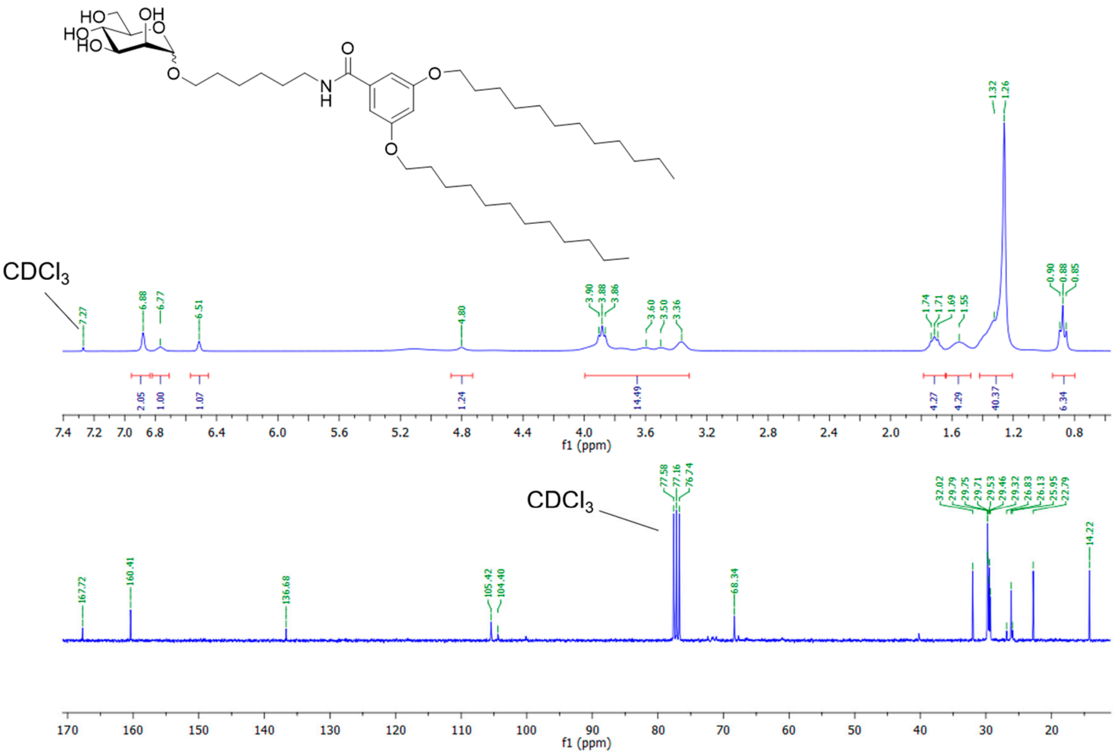

Figure S11. ^{1}H-NMR and ^{13}C-NMR (75 MHz, CDCl$_3$) of compound **23**.

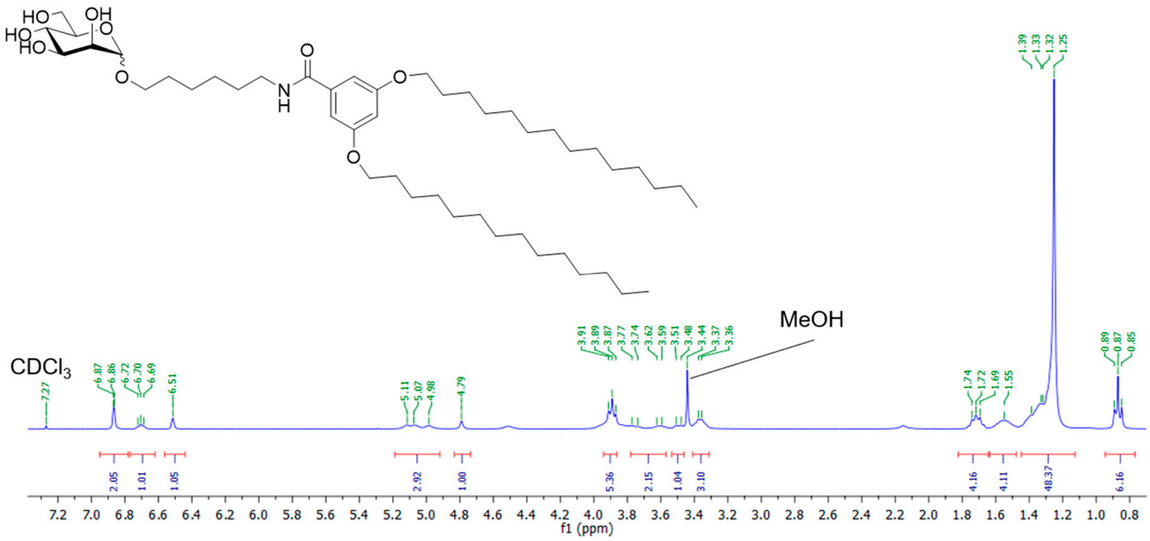

Figure S12. ^{1}H-NMR (300 MHz, $CDCl_3$) of compound **24.**

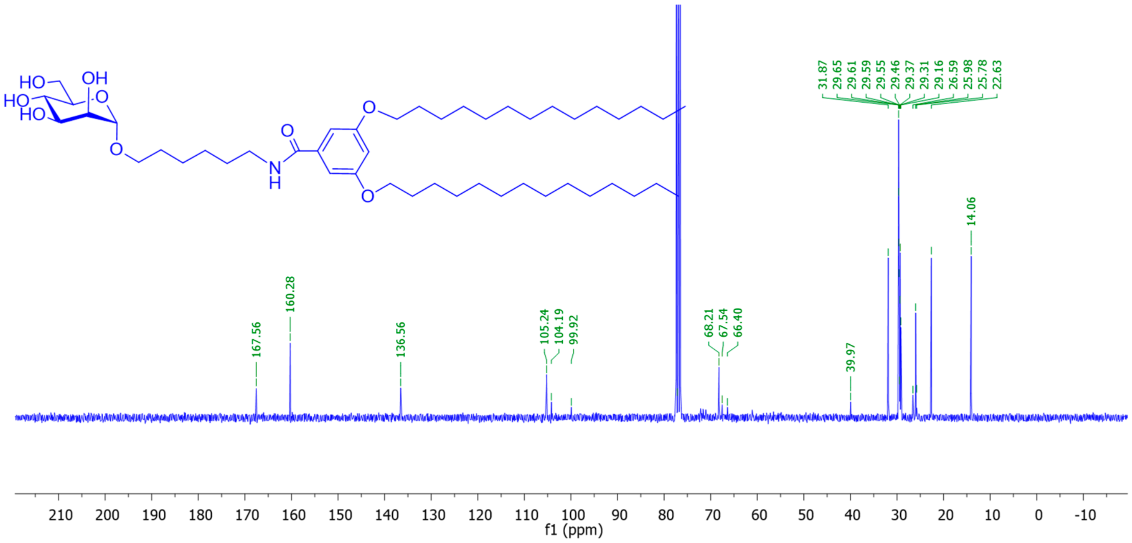

Figure S13. ^{13}C-NMR (300 MHz, $CDCl_3$) of compound **24**.

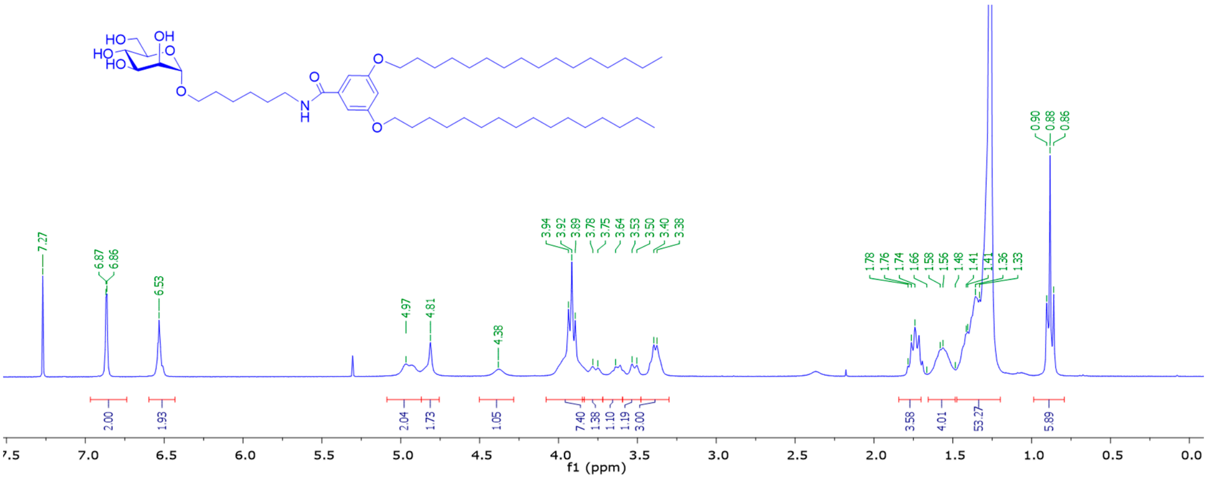

Figure S14. ^{1}H-NMR (300 MHz, $CDCl_3$) of compound **25**.

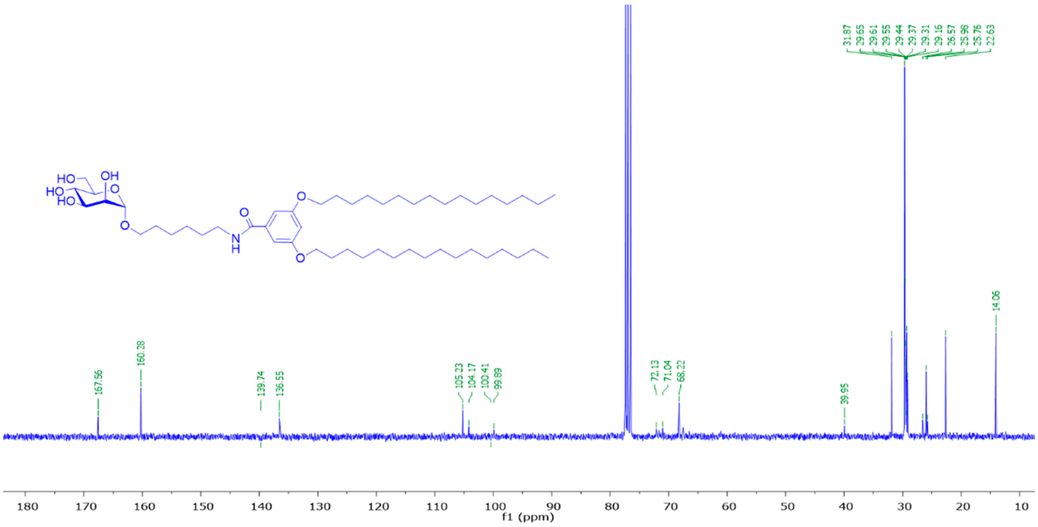

Figure S15. ^{13}C-NMR (75 MHz, $CDCl_3$) of compound **25**.
